# Supplementary figures and images for: A cortical network processes auditory error signals during human speech production to maintain fluency
Source: PLoS Biol. 2022 Feb 3;20(2):e3001493. doi: 10.1371/journal.pbio.3001493 (PMC8812883; doi:10.1371/journal.pbio.3001493)

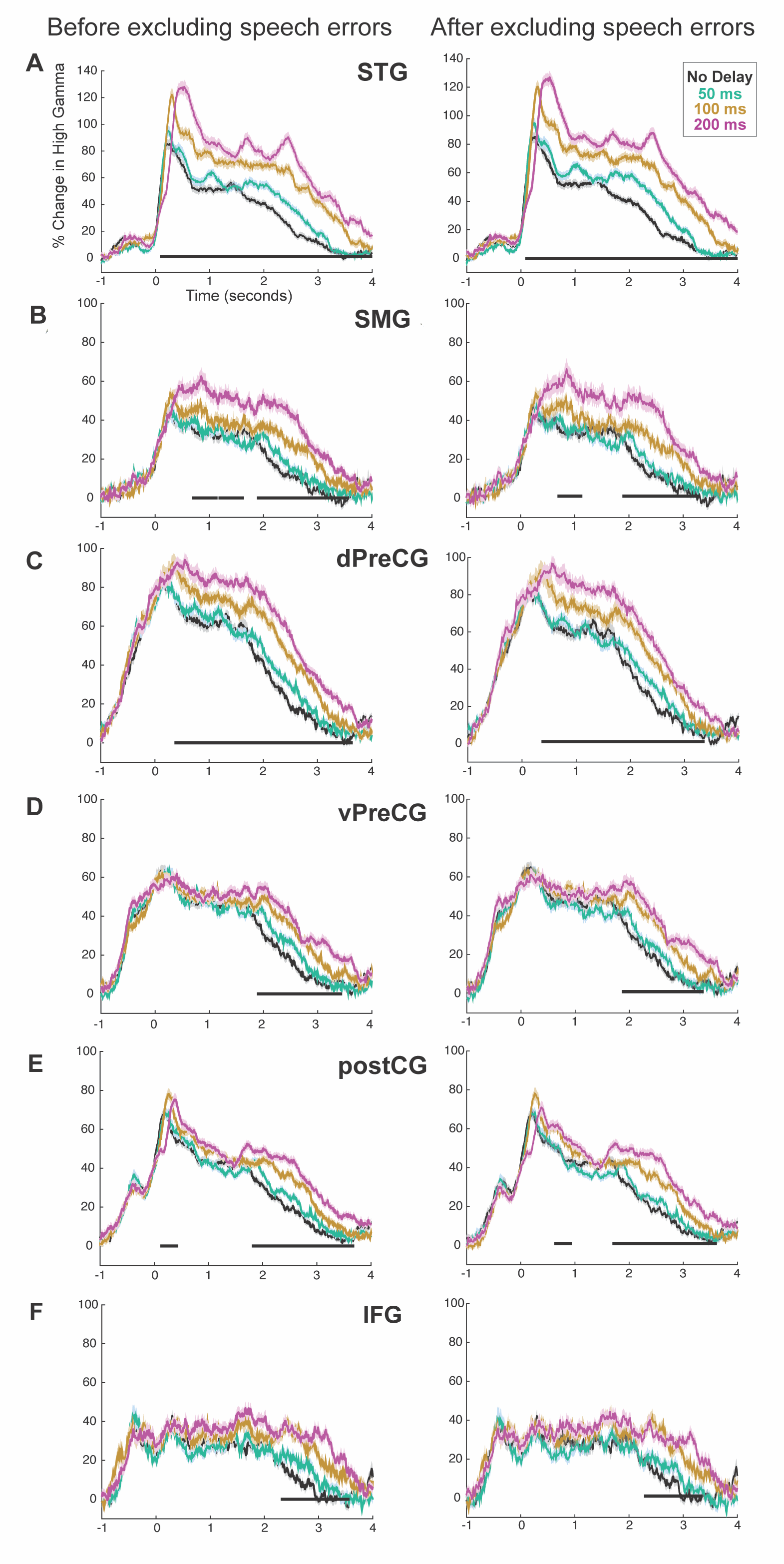

Supplement: S1 Fig — (A–F) High gamma responses for the DAF sentence reading in 6 different regions are shown. Responses before and after the exclusion of speech errors are shown in the left and right panels, respectively. Colors represent the various DAF conditions and shaded regions indicate SEM over trials. Black horizontal lines at the bottom of the graph indicate the time intervals when the neural responses diverged significantly across conditions. The underlying data can be found in https://github.com/flinkerlab/DelayedAuditoryFeedback. DAF, delayed auditory feedback; SEM, standard error of the mean. (TIF) [file pbio.3001493.s001.tif]

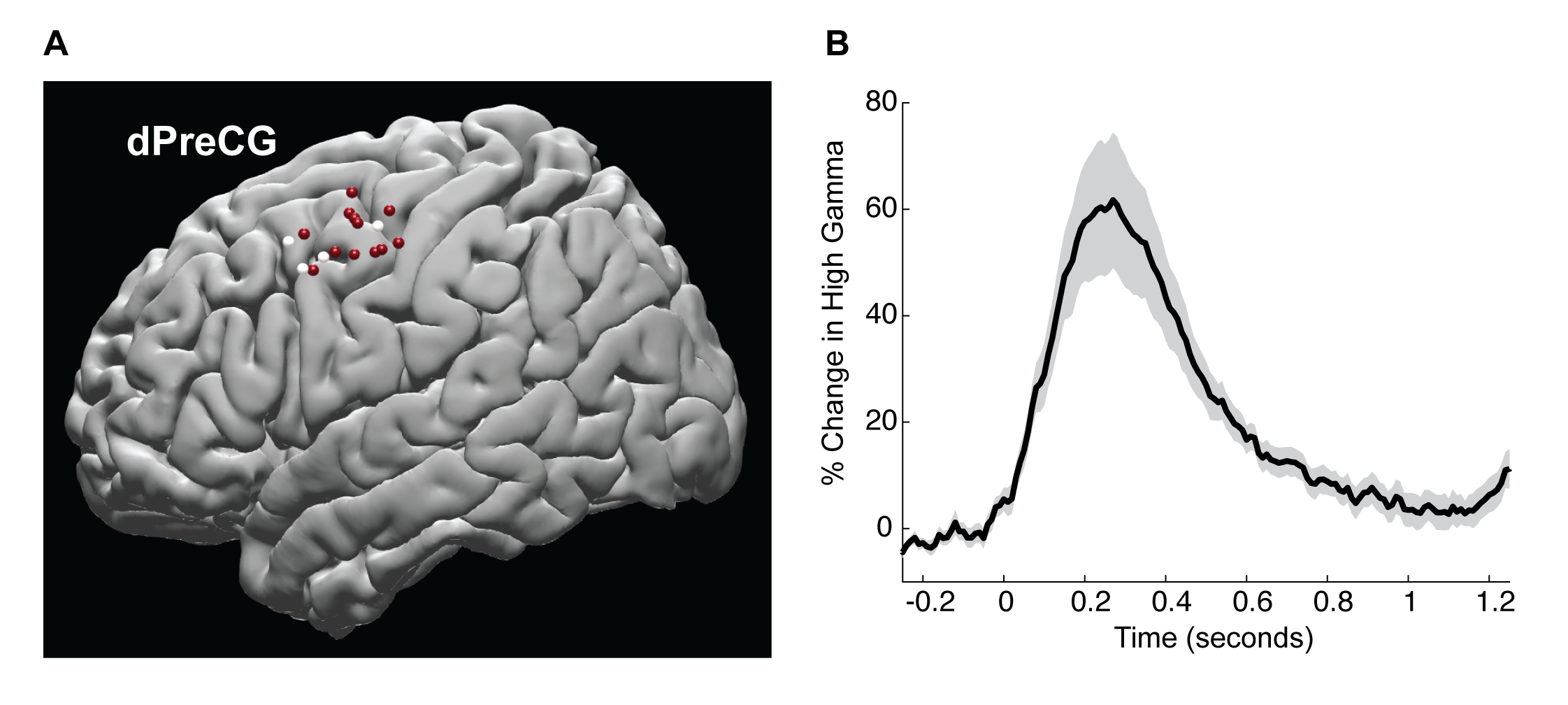

Supplement: S2 Fig — (A) Seventeen dPreCG electrodes in 5 participants are shown on a template brain. Electrodes that show a significant response during passive listening of words are shown in red. (B) Average high gamma responses across the dPreCG electrodes is shown. Shaded regions indicate SEM over trials. The underlying data can be found in https://github.com/flinkerlab/DelayedAuditoryFeedback. dPreCG, dorsal precentral gyrus; SEM, standard error of the mean. (TIF) [file pbio.3001493.s002.tif]

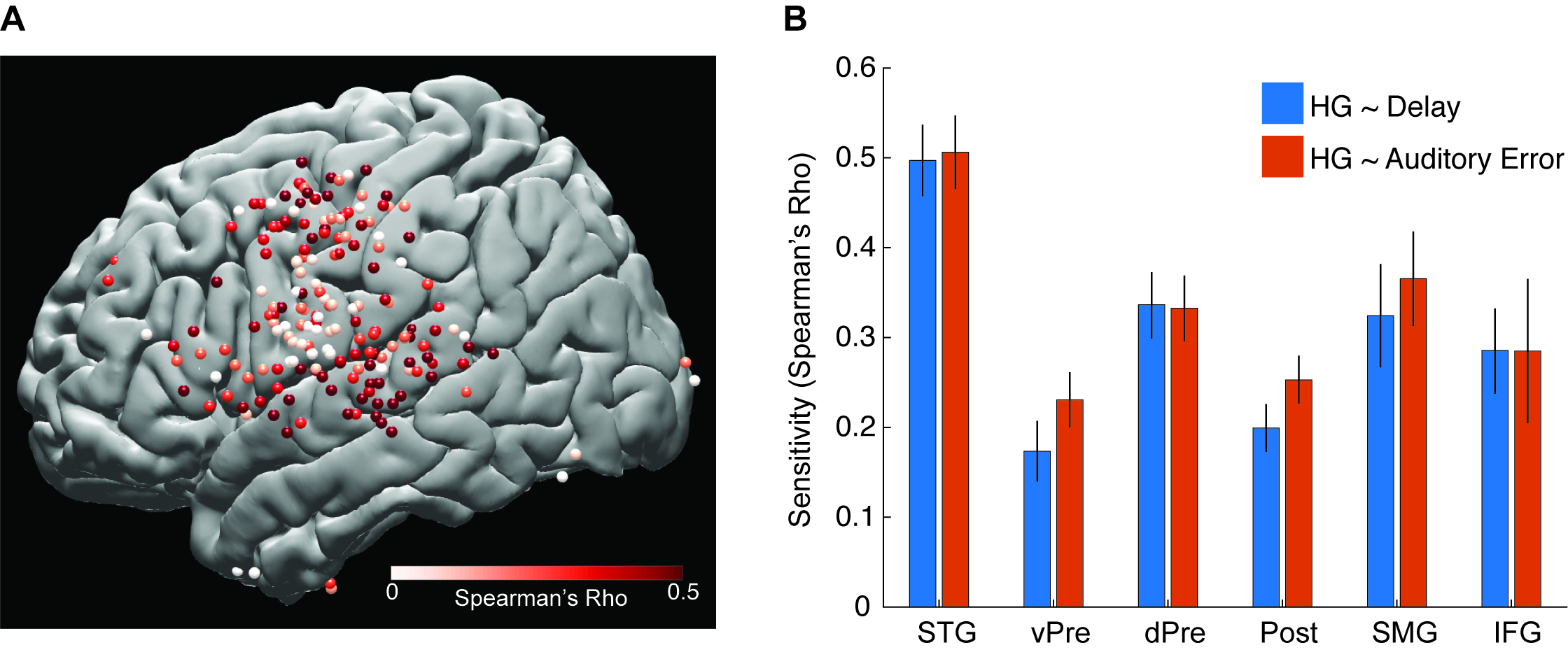

Supplement: S3 Fig — (A) Correlation between high gamma response and auditory error for each electrode is shown on the template brain. (B) Sensitivity to DAF measured as the correlation between high gamma response and delay versus the correlation between high gamma response and auditory error. The underlying data can be found in https://github.com/flinkerlab/DelayedAuditoryFeedback. DAF, delayed auditory feedback. (TIF) [file pbio.3001493.s003.tif]

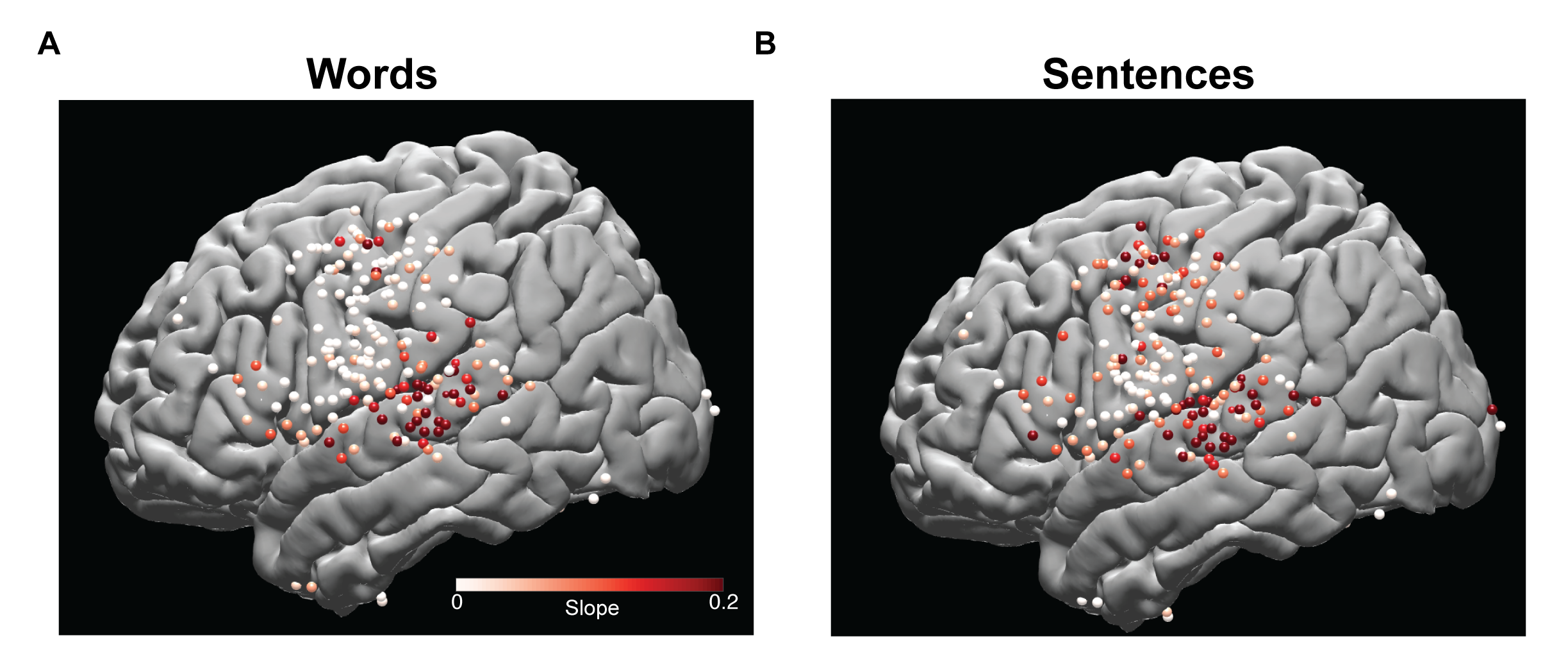

Supplement: S4 Fig — A linear model was fit to describe the relationship between neural response and delay condition. For each electrode, slope of the fitted line was used as measure of sensitivity to DAF and shown on a template brain for (A) word reading and (B) sentence reading tasks. The underlying data can be found in https://github.com/flinkerlab/DelayedAuditoryFeedback. DAF, delayed auditory feedback. (TIF) [file pbio.3001493.s004.tif]

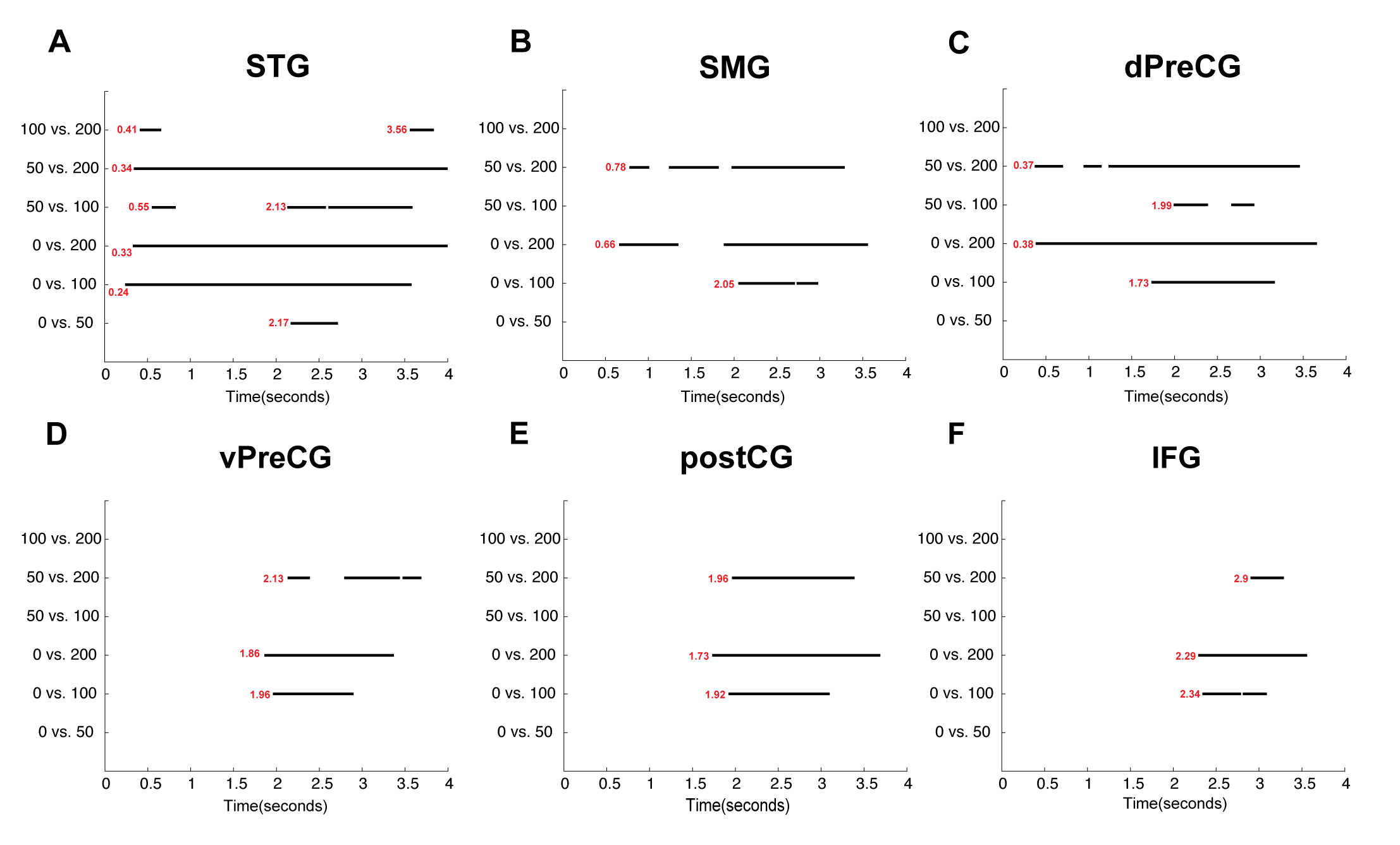

Supplement: S5 Fig — (A–F) Black horizontal lines indicate the time intervals when the neural response to the 2 delay conditions diverged significantly in 6 different regions of interest for at least 200 consecutive milliseconds (1-way ANOVA p<0.01 with FDR correction at q = 0.05). Divergence onset times are indicated with red text. The underlying data can be found in https://github.com/flinkerlab/DelayedAuditoryFeedback. FDR, false discovery rate. (TIF) [file pbio.3001493.s005.tif]

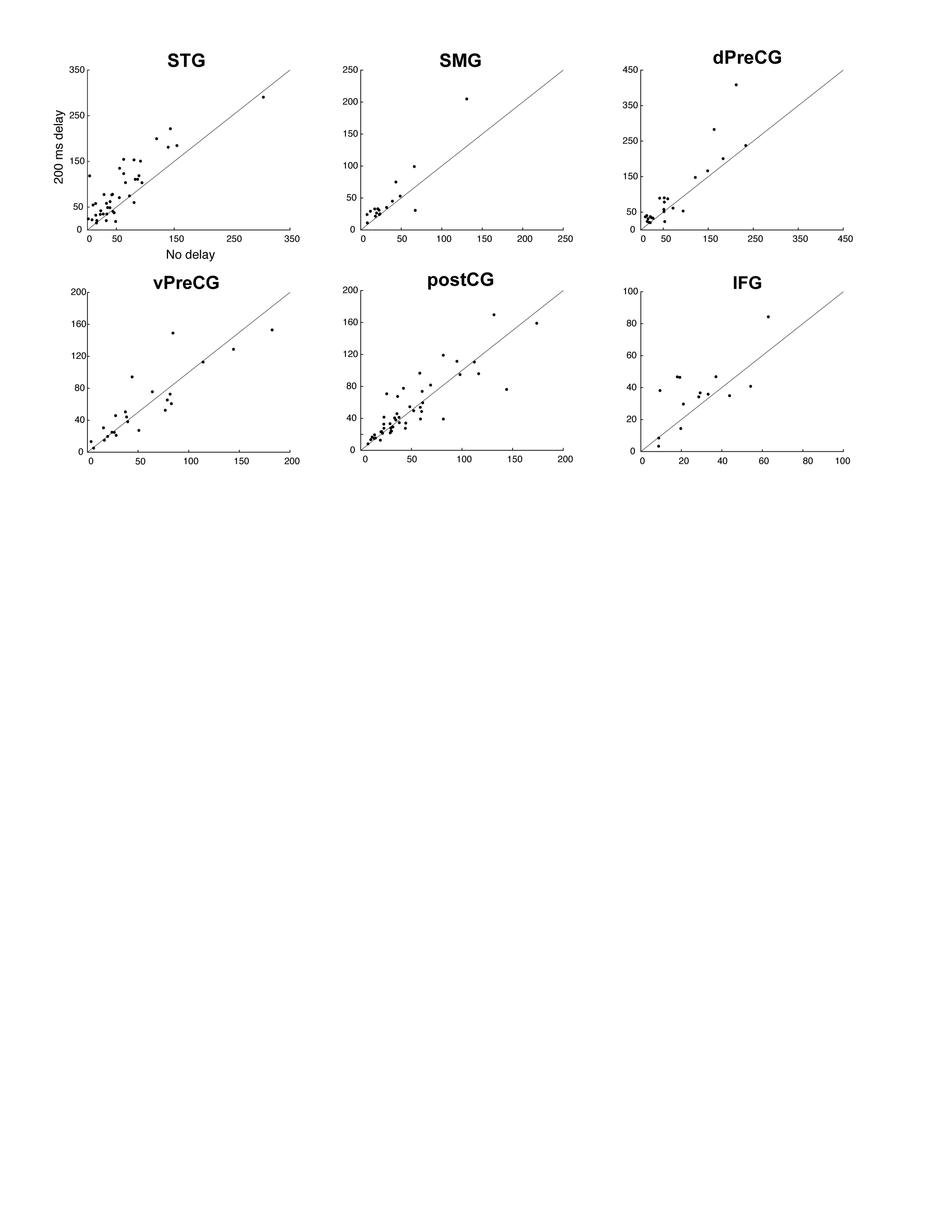

Supplement: S6 Fig — Scatter plots show the averaged high gamma responses between 0 and 2 seconds for “no delay” versus “200-millisecond delay” conditions for in each electrode (black circles) in different regions. Only STG, SMG, and dPreCG showed significantly larger neural responses for 200-millisecond delay condition (paired t test; STG: t = 5.6, p = 2 × 10^−6^, SMG: t = 2.2, p = 0.04, dPreCG: t = 2.43, p = 0.02, vPreCG: t = 0.39, p = 0.7, postCG: t = 0.86, p = 0.4, IFG: t = 2.03, p = 0.06). The underlying data can be found in https://github.com/flinkerlab/DelayedAuditoryFeedback. dPreCG, dorsal precentral gyrus; SMG, supramarginal gyrus; STG, superior temporal gyrus. (TIF) [file pbio.3001493.s006.tif]

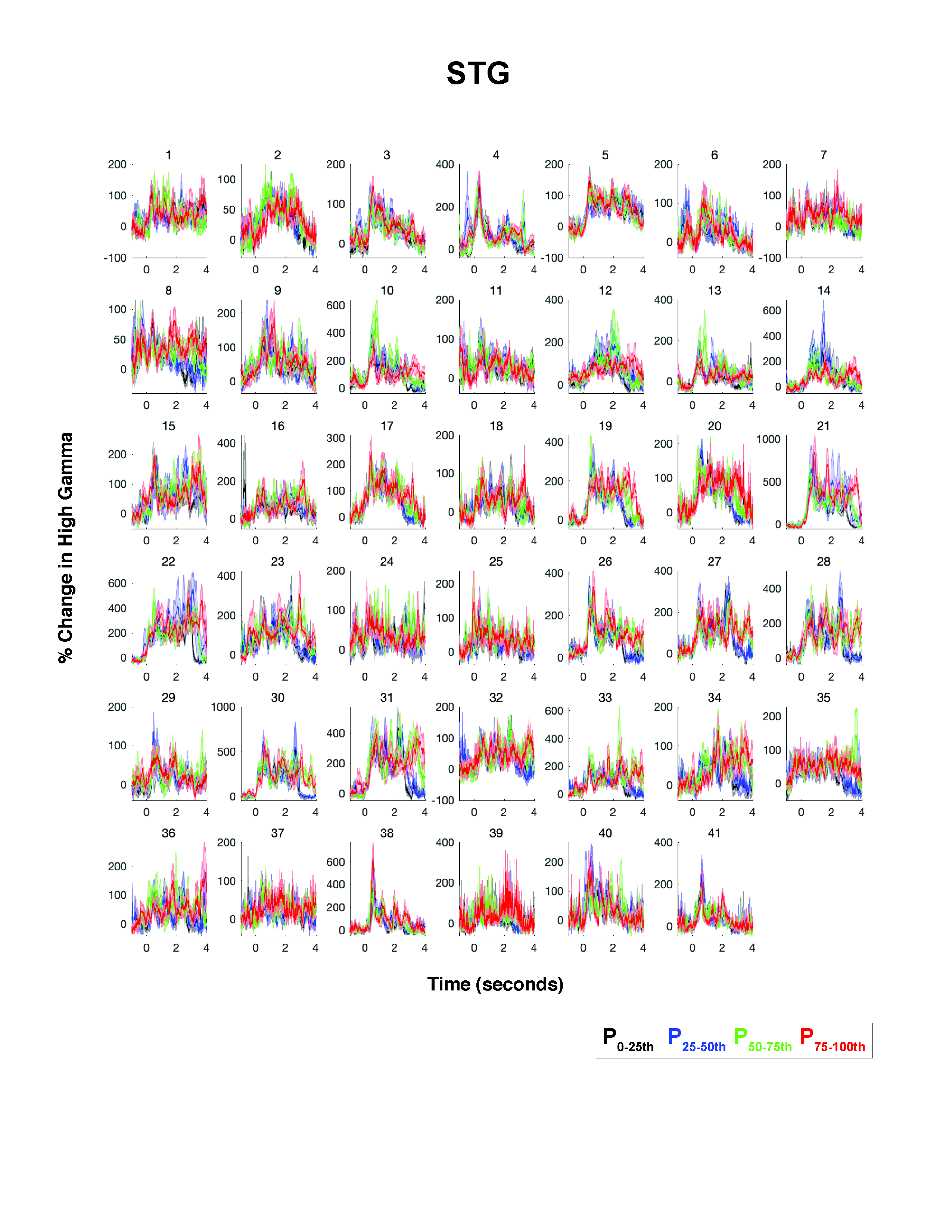

Supplement: S7 Fig — High gamma responses after 200-millisecond delay trials are split into 4 groups based on articulation duration are shown for each single electrode in STG. The underlying data can be found in https://github.com/flinkerlab/DelayedAuditoryFeedback. STG, superior temporal gyrus. (TIF) [file pbio.3001493.s007.tif]

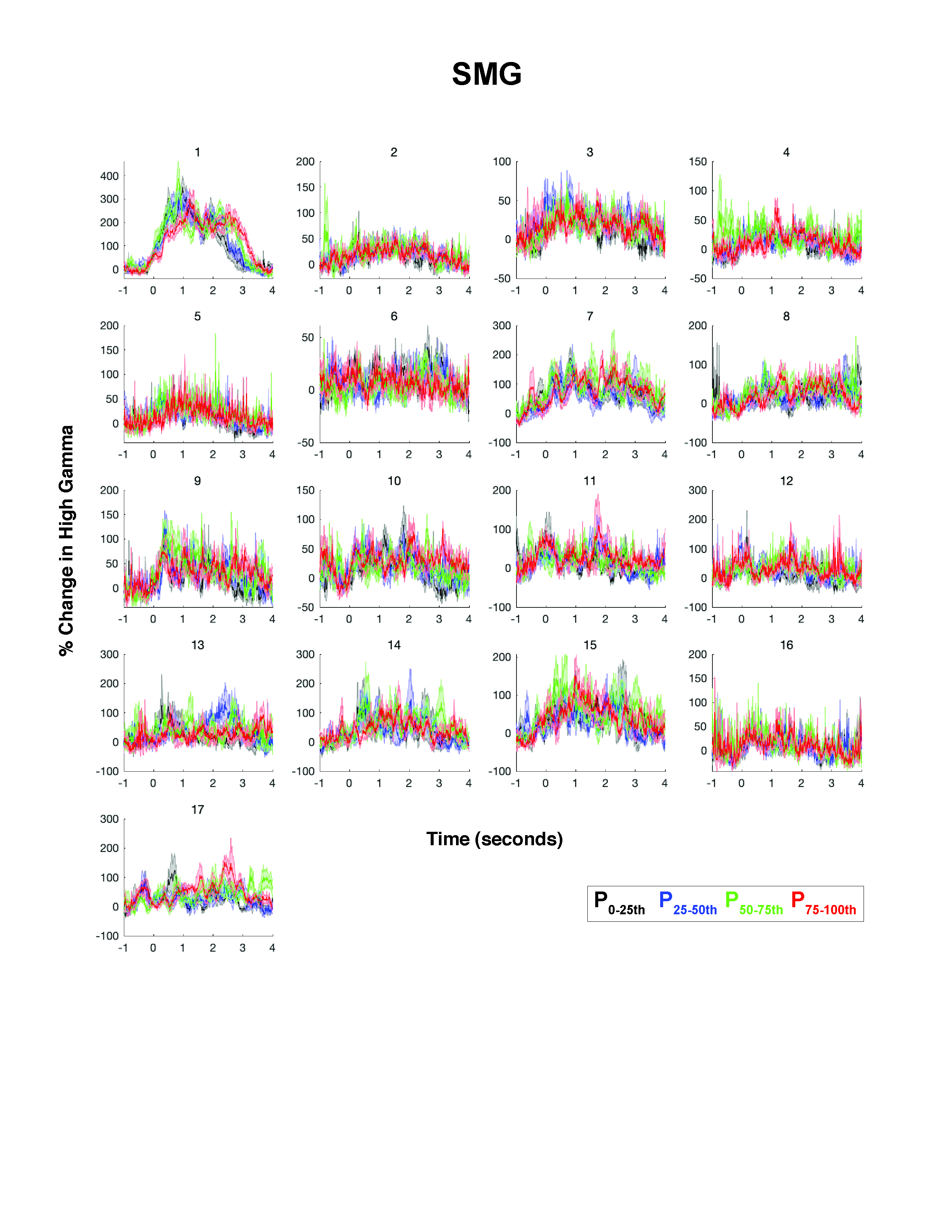

Supplement: S8 Fig — High gamma responses after 200-millisecond delay trials are split into 4 groups based on articulation duration are shown for each single electrode in SMG. The underlying data can be found in https://github.com/flinkerlab/DelayedAuditoryFeedback. SMG, supramarginal gyrus. (TIF) [file pbio.3001493.s008.tif]

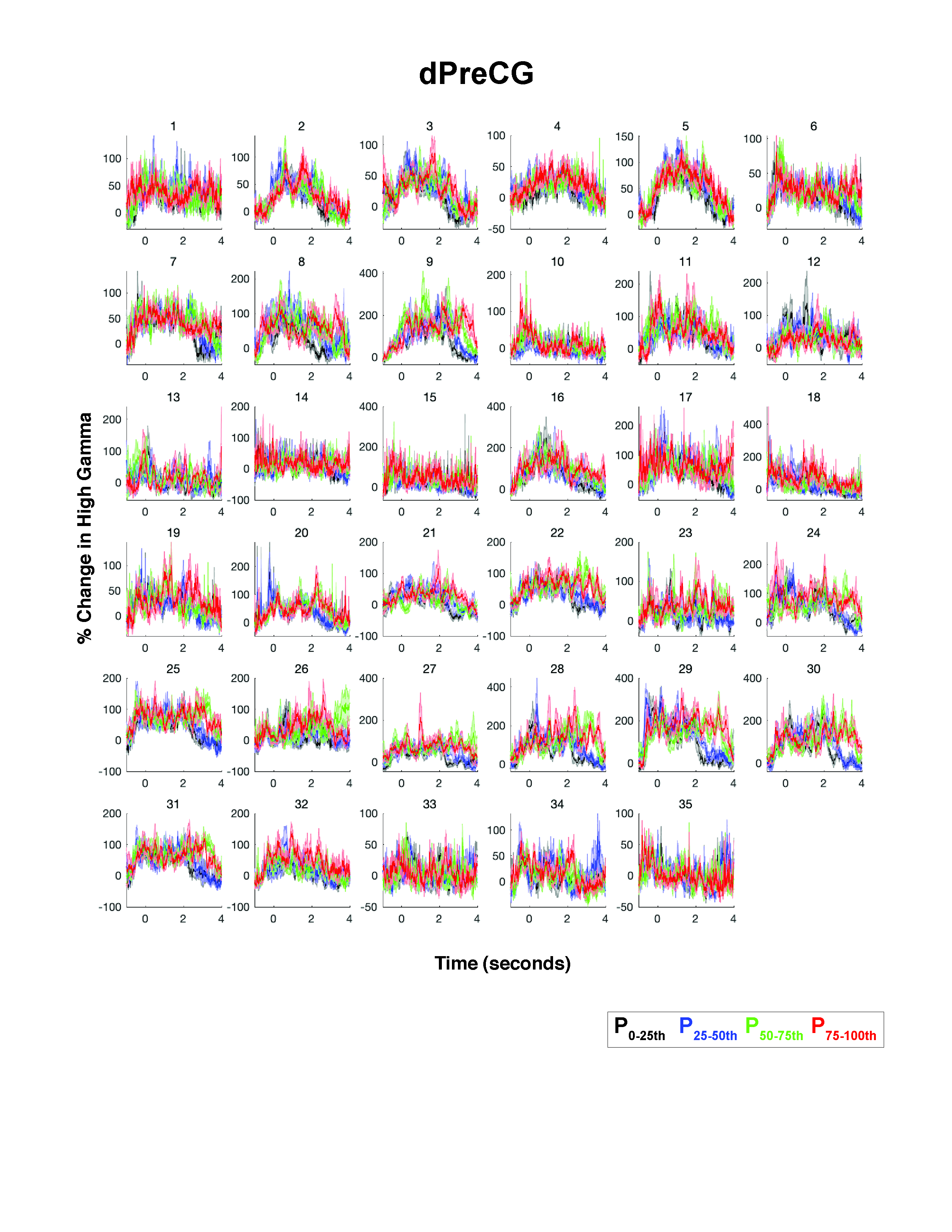

Supplement: S9 Fig — High gamma responses after 200-millisecond delay trials are split into 4 groups based on articulation duration are shown for each single electrode in dPreCG. The underlying data can be found in https://github.com/flinkerlab/DelayedAuditoryFeedback. dPreCG, dorsal precentral gyrus. (TIF) [file pbio.3001493.s009.tif]

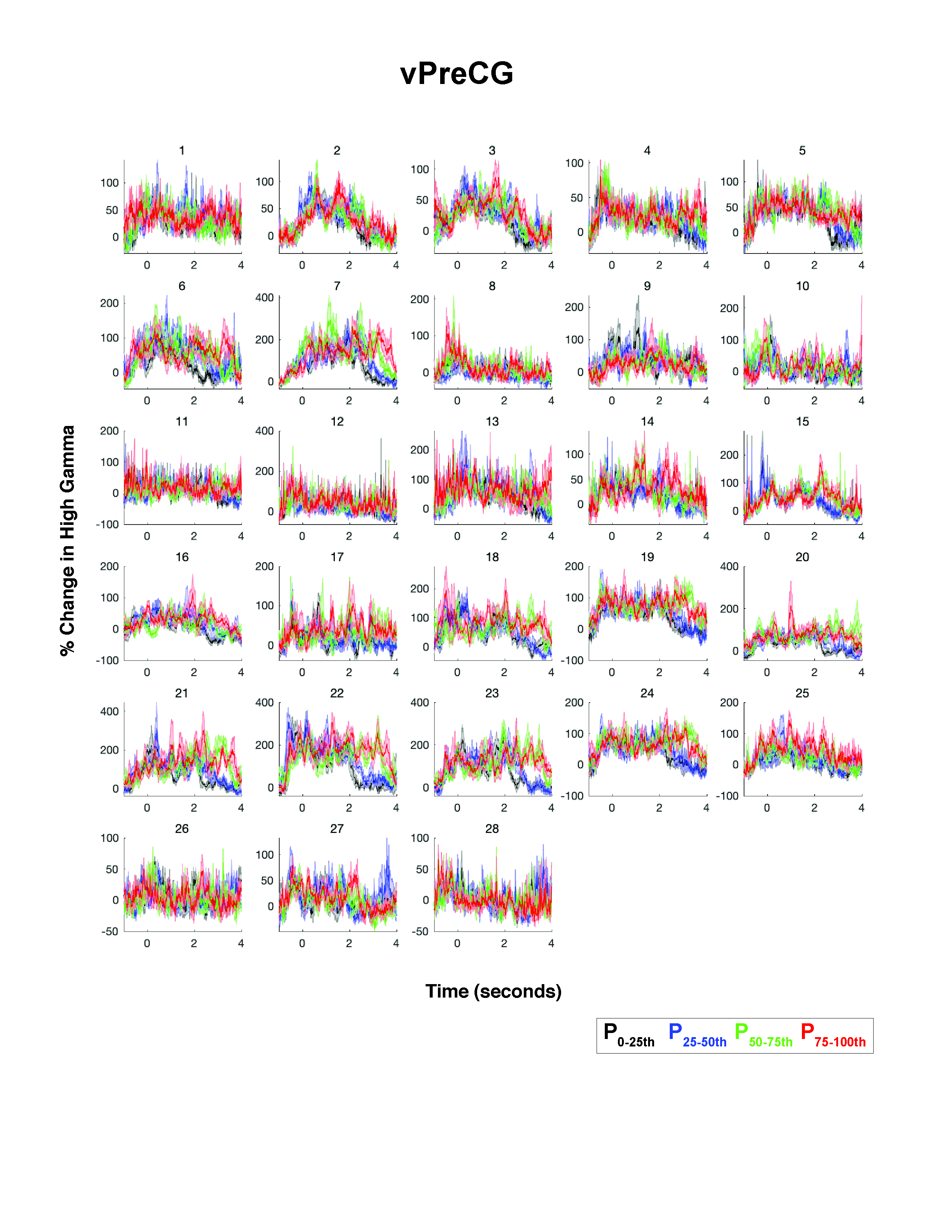

Supplement: S10 Fig — High gamma responses after 200-millisecond delay trials are split into 4 groups based on articulation duration are shown for each single electrode in vPreCG. The underlying data can be found in https://github.com/flinkerlab/DelayedAuditoryFeedback. vPreCG, ventral precentral gyrus. (TIF) [file pbio.3001493.s010.tif]

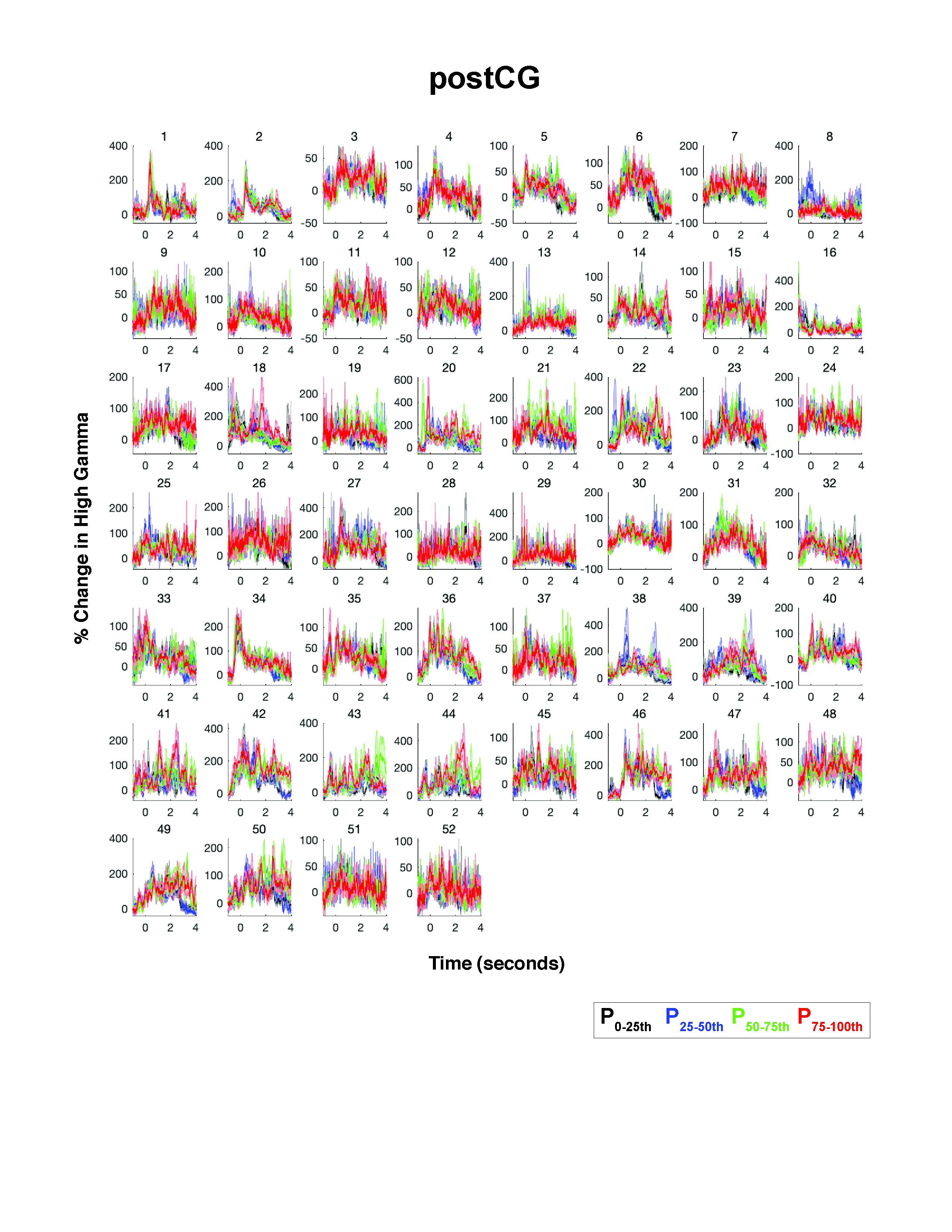

Supplement: S11 Fig — High gamma responses after 200-millisecond delay trials are split into 4 groups based on articulation duration are shown for each single electrode in postCG. The underlying data can be found in https://github.com/flinkerlab/DelayedAuditoryFeedback. postCG, postcentral gyrus. (TIF) [file pbio.3001493.s011.tif]

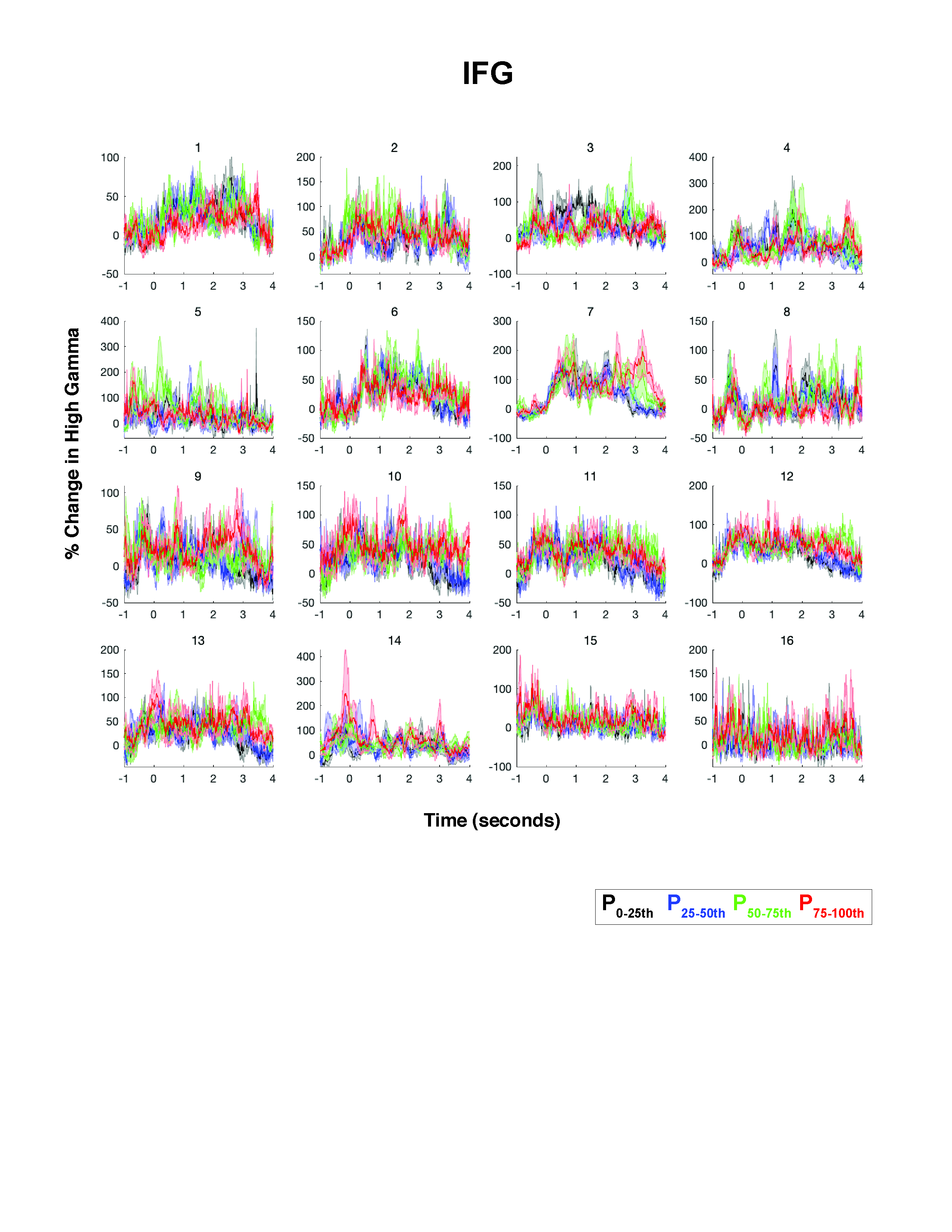

Supplement: S12 Fig — High gamma responses after 200-millisecond delay trials are split into 4 groups based on articulation duration are shown for each single electrode in IFG. The underlying data can be found in https://github.com/flinkerlab/DelayedAuditoryFeedback. IFG, inferior frontal gyrus. (TIF) [file pbio.3001493.s012.tif]
