# Supplementary material for: A cortical network processes auditory error signals during human speech production to maintain fluency
Source: PLoS Biol. 2022 Feb 3;20(2):e3001493. doi: 10.1371/journal.pbio.3001493 (PMC8812883; doi:10.1371/journal.pbio.3001493)
Supplement: S1 Text — (DOCX) [file pbio.3001493.s013.docx]

**Speech Error Analysis**

During the DAF sentence-reading task, both the articulation duration and the neural response amplitudes were significantly increased. To investigate whether these effects were driven by specific speech errors, we performed a detailed analysis. Two independent evaluators with speech language pathology training examined the audio recordings of subjects for different kinds of speech errors as described by Chon et al. 2013. These errors were grouped in three categories including Speech Errors (SE: sound substitutions, omissions and additions), Stutter Like Dysfluencies (SLD: prolongations, repetitions, pauses > 250ms, blocks and voice tremor) and Other Dysfluencies (OD: revisions, phrase repetitions, phoneme errors and interjections). We excluded a total of 206 trials (12%) that both evaluators reported to contain either SE (1%), SLD (7%) or OD (4%) errors. We demonstrated the neural responses in different brain regions after excluding the trials with speech errors (**S1 Fig**). We performed a one-way ANOVA to determine the time intervals when the neural responses diverged significantly for at least 200 consecutive milliseconds (p<0.001, FDR corrected at q=0.05). Excluding trials with speech errors yielded almost identical result to our original findings with early and strong response enhancement of neural responses to DAF in STG and dPreCG.

**References:**

Chon, H., Kraft, S. J., Zhang, J., Loucks, T., & Ambrose, N. G. (2013). Individual variability in delayed auditory feedback effects on speech fluency and rate in normally fluent adults. *Journal of speech, language, and hearing research: JSLHR*, *56*(2), 489–504.
