# Supplementary material for: A cortical network processes auditory error signals during human speech production to maintain fluency
Source: PLoS Biol. 2022 Feb 3;20(2):e3001493. doi: 10.1371/journal.pbio.3001493 (PMC8812883; doi:10.1371/journal.pbio.3001493)
Supplement: S2 Text — (DOCX) [file pbio.3001493.s014.docx]

**Passive Listening Task**

dPreCG and the surrounding cortex is a functionally complex region, which has been shown to be involved in both motor and sensory aspects of speech processing (Cheung et al. 2016, Dichter et al. 2018). To further examine the involvement of dPreCG electrodes in auditory processing, we analyzed data from an additional passive word-listening task with 5 of our subjects, in which they were presented with speech stimuli recorded by a female speaker. The majority of dPreCG electrodes in these 5 subjects (13 out of 17 electrodes shown in red on the template brain) showed a significant response increase during passive listening (0-500 ms after speech onset) with respect to baseline (-500 to -100 ms before speech onset; paired t-test, p<0.01). We show the average high gamma response across these electrodes (**S2 Fig**), which demonstrates the involvement of dPreCG in auditory processing.

**References:**

Cheung, C., Hamiton, L. S., Johnson, K., & Chang, E. F. (2016). The auditory representation of speech sounds in human motor cortex. *eLife*, *5*, e12577.

Dichter, B. K., Breshears, J. D., Leonard, M. K., & Chang, E. F. (2018). The Control of Vocal Pitch in Human Laryngeal Motor Cortex. *Cell*, *174*(1), 21–31.e9.
