# Supplementary material for: A cortical network processes auditory error signals during human speech production to maintain fluency
Source: PLoS Biol. 2022 Feb 3;20(2):e3001493. doi: 10.1371/journal.pbio.3001493 (PMC8812883; doi:10.1371/journal.pbio.3001493)
Supplement: S3 Text — (DOCX) [file pbio.3001493.s015.docx]

**Auditory Error Calculation**

In a DAF paradigm, the auditory error can be represented by the difference between the target and the feedback acoustic signal caused by the time delay. We calculated this mismatch as the absolute difference between the original and time-shifted (shifted by 50, 100 or 200 ms) speech spectrograms. We then correlated this auditory error with neural activity to quantify the sensitivity to DAF in each electrode. We obtained very similar results to when we correlated feedback delay with neural activity (**S3 Fig**).
