# Supplementary material for: A cortical network processes auditory error signals during human speech production to maintain fluency
Source: PLoS Biol. 2022 Feb 3;20(2):e3001493. doi: 10.1371/journal.pbio.3001493 (PMC8812883; doi:10.1371/journal.pbio.3001493)
Supplement: S4 Text — DAF, delayed auditory feedback. (DOCX) [file pbio.3001493.s016.docx]

**Linear regression analysis to calculate sensitivity to DAF**

To quantify sensitivity to DAF, we originally calculated the correlation between neural activity and delay condition for each electrode. We performed an additional linear regression analysis by sorting the trials with respect to delay condition (no delay, 50, 100 and 200), calculating the mean high gamma activity for each trial and fitting a regression across trials for each electrode. As a measure of sensitivity, we showed the slope values for each electrode for the word-reading and the sentence-reading task (**S4 Fig**). The anatomical distribution of slope values showed a strong resemblance to the distribution of Spearman correlation values (**Fig 3E-3F**). Moreover, sensitivity to DAF measured using slope values was also significantly larger for sentence-reading task compared to word-reading task (paired t-test: t=2.3, p=0.02).
