# Supplementary material for: A cortical network processes auditory error signals during human speech production to maintain fluency
Source: PLoS Biol. 2022 Feb 3;20(2):e3001493. doi: 10.1371/journal.pbio.3001493 (PMC8812883; doi:10.1371/journal.pbio.3001493)
Supplement: S5 Text — (DOCX) [file pbio.3001493.s017.docx]

**Pairwise Comparison of Neural Responses:**

We performed post hoc pairwise comparisons (FDR corrected at p = 0.05 and p<0.01) to test whether the divergence onset was different for different delays (**S5 Fig**). For STG, divergence onset occurred earliest for no delay vs. 100, at 240 ms after speech onset. For no delay vs. 200, divergence occurred at 330 ms. And for no delay vs. 50, divergence occurred much later at 2.17 s. When we compared the no delay versus 200 ms delay conditions, the onset of divergence followed the same order as when we compared all four conditions: divergence onset occurred the earliest in the STG at 330 ms, then in dPreCG at 380 ms, in SMG at 660 ms, in postCG at 1.73 s, in vPreCG at 1.86 s and finally in IFG at 2.29 s.
