# Supplementary material for: A cortical network processes auditory error signals during human speech production to maintain fluency
Source: PLoS Biol. 2022 Feb 3;20(2):e3001493. doi: 10.1371/journal.pbio.3001493 (PMC8812883; doi:10.1371/journal.pbio.3001493)
Supplement: Attachment — Submitted filename: Response to Reviewers.pdf [file pbio.3001493.s018.pdf]

We would like to thank the editor and reviewers for their thoughtful comments which greatly strengthens our manuscript. To address their concerns, we now provide additional ECoG data obtained during a passive listening task and a regular word-reading task (without DAF). Moreover, we include new analyses in which we control for articulatory aspects of the responses to eliminate the possibility that neural response enhancement is due to longer articulation duration. We also provide a detailed speech error analysis performed by two independent evaluators to demonstrate that neural response enhancement to DAF is not caused by speech errors. In this resubmission, we now include two new main figures (**Fig 6-7**) and a Supporting Information document with five supplementary figures. Below, we provide a point by point response to reviewers:

Reviewer #1: This study examined cortical neural signals during speech production with delayed-auditory feedback. The question that the authors aim to address is an important one and the manuscript reported interesting data under several experimental conditions and behavioral responses. However, the current report lacks necessary analyses to fully support a novel and significant function of the observed activity (e.g. in the frontal region). While the activity in dorsal precentral gyrus is interesting, it is unclear what this activity represents in terms of auditory error or articulatory control.

<u>Main concerns:</u>
(1) In sentence reading, articulation duration is found to be increased with delay (p.7, Fig. 3). How is this analysis done? Were data included here only the correct trials or was there any speech error in these productions? It is important to delineate the types of production in this condition since it is related to activities observed in the frontal cortex (or Cluster 2). If errors are included in these trials, please give examples where subjects made errors, pauses, or restarted speech.
We originally included all the trials in our analysis, as the patients did not make any speech errors in the word-reading task (where we performed clustering) and made few errors in the sentence-reading task. However, we understand the reviewer's concern and to specifically address it, we solicited two independent speech evaluators to perform a detailed analysis of errors. Two evaluators with speech language pathology training (NYU Steinhardt Speech Language Pathology), examined and scored the audio recordings of subjects for different kinds of speech errors as described by Chon, et al. 2013. These errors were categorized as Speech Errors (SE: i.e. sound substitutions, omissions and additions), Stutter Like Dysfluencies (SLD: i.e. prolongations, repetitions, pauses > 250ms, blocks and voice tremor) and Other Dysfluencies (OD: i.e. revisions, phrase repetitions, phoneme errors and interjections). We excluded a total of 206 trials (12%) that both evaluators reported to contain either SE (1%), SLD (7%) or OD (4%) errors. In the below figure, we demonstrate the neural responses in different brain regions before and after excluding the trials with speech errors. Horizontal black lines indicate the intervals when the neural responses diverged significantly for at least 200 ms (one-way ANOVA: $p<0.001$, FDR corrected at $q=0.05$). Excluding trials with speech errors yielded almost identical results to our original findings with early and strong response enhancement of neural responses to DAF in STG and dPreCG. We now include these new results in the *Supporting Information* (**S1 Fig.**).

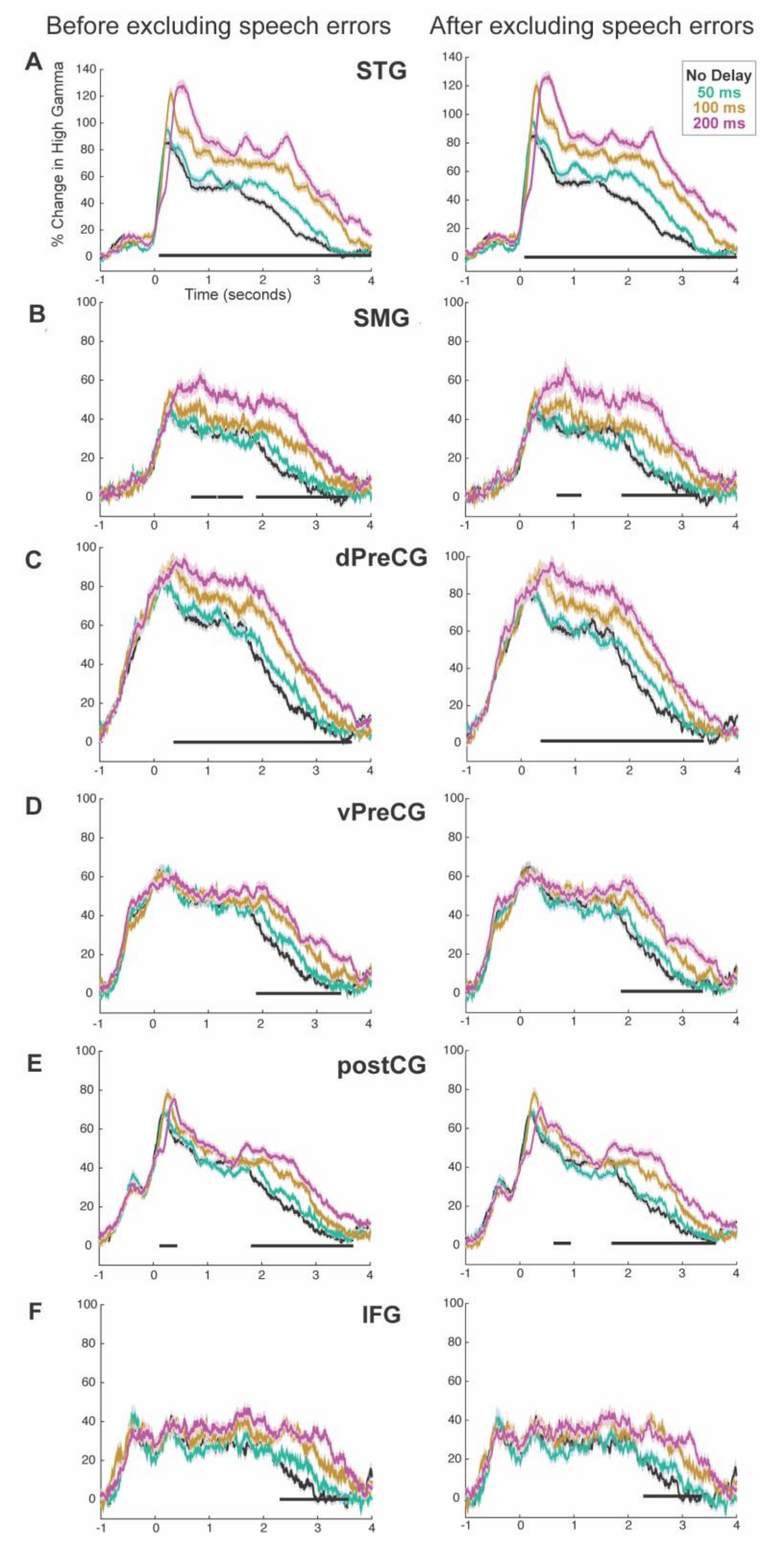

S1 Fig. Neural responses before and after excluding trials with speech errors

(2) Previous studies have shown that dorsal part of the sensorimotor cortex has auditory responses to speech. There is also a supposed laryngeal motor region close to what the authors describe as dPreCG. The overlapping location of these activities complicates the interpretation in this study. Without careful analysis, it is hard to say whether the increased activity in dPreCG during longer delay is due to auditory responses/error signals relayed from STG area or simply that the subject is making more efforts to speak (e.g. difference in articulation; restarting production after frequent pauses). Therefore, the claim about dPreCG is lacking support.
We agree with the reviewer that dPreCG and the surrounding cortex is a functionally complex region, which has been shown to be involved in both motor and sensory aspects of speech processing. To further examine the involvement of dPreCG electrodes in auditory processing, we analyzed data from an additional passive word-listening task with 5 of our subjects, in which they were presented with auditory speech stimuli (female speaker recording). The majority of dPreCG electrodes in these 5 subjects (13 out of 17 electrodes shown in red on the template brain) showed a significant response increase during passive listening (0-500 ms after speech onset) with respect to baseline (-500 to -100 ms before speech onset; paired t-test, $p<0.01$). The average high gamma response across these electrodes is shown below, demonstrating the involvement of dPreCG in auditory processing. We now include these new results in the *Supporting Information* (**S2 Fig.**).

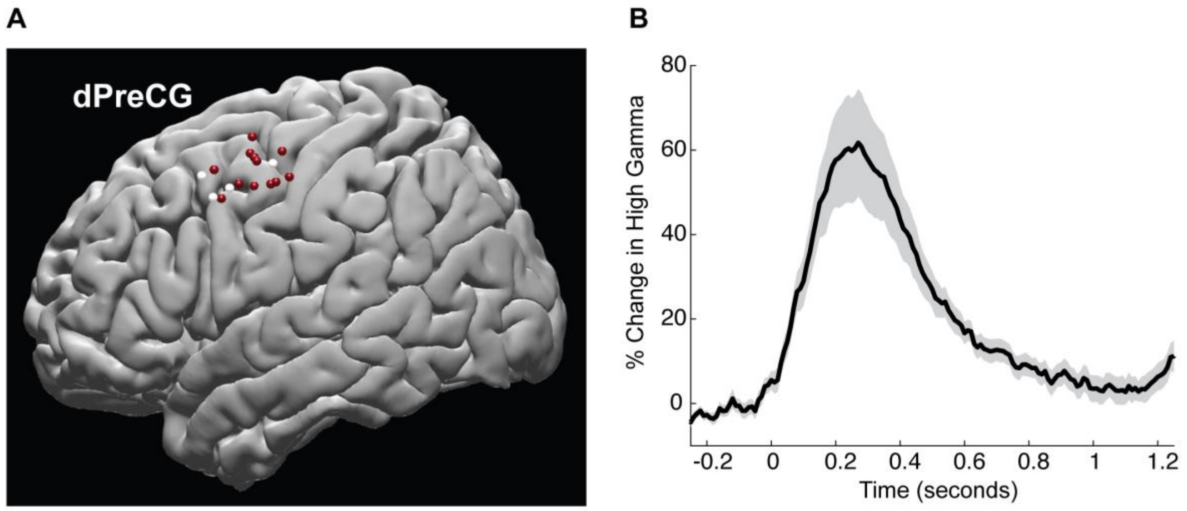

S2 Fig. Neural responses to a passive listening task in PreCG

In addition to this auditory response, we provide results from several more analyses which argue against motor-articulatory related neural response enhancement in dPreCG. In our response to Reviewer 3, we identify no delay and 200 ms DAF trials that match in articulation duration and compared the neural responses for these trial pairs. We found that neural responses were still enhanced in dPreCG for DAF even when articulation duration was controlled. Next, in response to Reviewer 2 (Question 5), we analyzed only the 200 ms DAF condition and split trials into 4 groups based on articulation duration (25, 50, 75 and 100 percentiles). When we compared the neural responses for different articulation durations, we did not find a significant neural response enhancement in dPreCG for longer articulation durations. Taken together, these results strongly suggest that response enhancement in dPreCG during DAF is not motor-articulatory related but rather indicates auditory error processing in this region.

(3) The mismatch between sensory feedback and efference copy is not well quantified. The author assumed that the magnitude of auditory error increases with the feedback delay, which seems reasonable in general. However, in previous studies with altered feedback in fundamental frequency or formants, it is quite clear what the production target is and therefore the auditory error can be calculated. In the delayed auditory feedback, the authors need to either refer to literature or perform analysis to show how the specific delay parameters (50ms, 100ms, 200ms) induce mismatch (e.g. overlapping syllables, pitch contour, etc). One correlation this may have on neural signals is that: 200ms delay seems to cause a delay in the response onset in auditory areas (Fig. 1D, Fig. 2C, Fig. 3C).
As per the reviewer's suggestion, we performed an additional analysis to calculate the actual auditory error. In a DAF paradigm, the auditory mismatch can be represented by the difference between the target and the feedback acoustic signal caused by the time delay. We calculated this mismatch as the absolute difference between the original and time-shifted speech spectrograms. When we correlated this auditory error with neural activity for each electrode, we obtained very similar results to when we correlated feedback delay with neural activity. We now include a new supplementary figure (shown below) summarizing the results of this analysis. The template brain shows the correlation between auditory error and high gamma response for each electrode. The bar graphs show the average correlation values for different regions of interest. As demonstrated by the bar graphs, correlating the high gamma response with the auditory error yielded similar results to correlating it with the delay. We now include these new results in the *Supporting Information* (**S3 Fig.**).

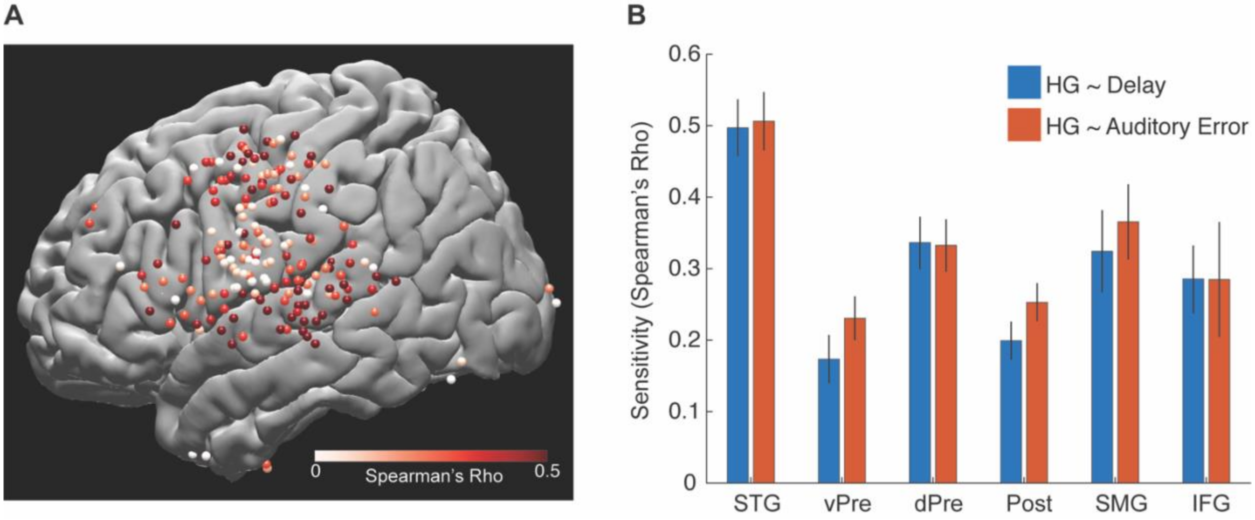

S3 Fig. Sensitivity to DAF measured as the correlation between neural response and auditory error

(4) It is known that STG area shows speech-induced suppression compared to passively listening to the playback of one's own speech. Have the authors investigated this for the dPreCG region?
With 5 of our subjects, we ran two additional tasks: a passive word-listening task and a word-reading task. In the passive listening task, subjects were presented with auditory speech stimuli (female voice recording). While our patients did not listen to the playback of their own speech, we calculated and demonstrated suppression following previously reported studies in the STG (Flinker et al. 2010). Thirteen out of seventeen (76.5%) dPreCG electrodes in our five subjects showed significant response increase during passive listening (as shown in the response to Question 2, **S2 Fig.**). We examined whether the neural activity in these electrodes is suppressed

when subjects produced speech in the word-reading task. The figure below shows the high gamma responses for the word-reading and passive listening tasks, and time 0 indicates the speech onset. We found that for the word-reading task, neural activity peaked approximately 300 ms prior to speech onset suggesting a preparatory response. We also observed a smaller secondary peak immediately after speech onset, which could be a response to the feedback of one's own speech. This secondary peak during word-reading was smaller in amplitude compared to the peak during passive listening, which provides evidence for speech-induced suppression. However, it's difficult to directly compare these two responses in dPreCG, since the observed activity during word-reading is a combination of motor and auditory responses. While in the STG, such comparison is more straightforward since the observed responses are mainly auditory and occur in the same time period following speech onset.

Neural Response in dPreCG during Passive Listening and Word Reading

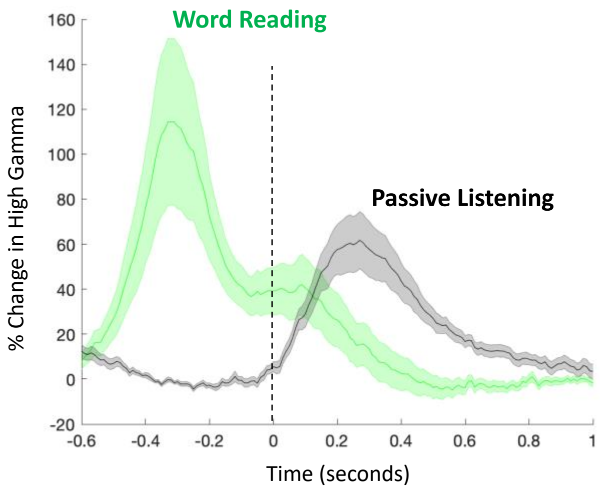

<u>Minor issues:</u>

(1) In abstract (line. 9) What do "these neural markers" refer to? Please specify. The authors mentioned both efference copy and auditory error in the previous sentences but I'm not sure if the data can suggest much on efference copy.
We now replaced "these neural markers" with "auditory error signal" and wrote in the abstract: "We were able to localize the auditory error signal using electrocorticographic recordings from neurosurgical subjects during a delayed auditory feedback (DAF) paradigm."

(2) The statement at the end of the abstract lacks support from the data. Signals in dPreCG seem to be correlated with the amount of delay (e.g. related to error), but little is shown regarding "internal speech estimates" or "efference copy". Please revise.
We now changed the statement at the end of the abstract and wrote: "These results suggest that dPreCG plays an essential role in processing auditory error signals during speech production to maintain fluency."

(3) Page 9, 2nd paragraph: the authors attribute the larger sensitivity to the articulation of longer speech segments. However, this could be due to the fact that more errors are being detected in the long speech segments and the sensitivity is not associated with articulation per se. Please

discuss all possible interpretations.
We agree with the reviewer that the mentioned statement is not an accurate interpretation of our results. To clarify this statement, we now write: “Moreover, several sites such as dPreCG and IFG showed increased sensitivity in the sentence-reading task. This result suggests that articulating longer and more complex stimuli during DAF not only elicits a stronger behavioral response but also results in stronger neural response enhancement across auditory and motor regions and engages a larger brain network uniquely recruiting additional frontal regions.”

Reviewer #2: The authors describe a series of experiment using human intracranial recording (ECoG) to examine feedback error detection during human speech. Unlike most recent work on this issue, the authors return to a much older methods of altering speech auditory feedback, delays, a manipulation that has seen much less recent use. They found an increase in activity of STG during delayed feedback that scaled with the delay, consistent with the error signal hypothesis. They also found an area of dorsal pre-central gyrus (which I presume to be dorsal pre-motor cortex) that also exhibited similar activity. Interestingly, the effect in the dPreCG is only really detectable during sentence rather than single word production.
Overall I find the work interesting and well done. It fits in well with increasing interest in the sensorimotor control of speech, and role of efference copy sensory prediction in motor control more broadly. I have a few questions and clarifications that I hope will help strengthen this very exciting work. I detail these below.

1. Was a purely sensory 'playback' experiment done (i.e. presenting the recorded sounds back to subjects when they are passively listening). This would help exclude the possibility of a purely sensory explanation for observed activity, though I think that would be an unlikely alternative hypothesis.
While our subjects did not listen to the playback of their own speech in a passive listening experiment, five of our subjects performed an additional passive word-listening task (female speaker recording). A majority (76.5%) of dPreCG electrodes in these 5 subjects (13 out of 17 electrodes shown in red on the template brain) showed significant response increase during passive listening (0-500 ms after speech onset) with respect to baseline (-500 to -100 ms before speech onset; unpaired t-test, $p<0.01$). The average high gamma response across these electrodes is shown below and is also discussed in response to Reviewer 1’s comments (Reviewer 1-Question 2). It is note-worthy that in addition to this auditory response, the dPreCG electrodes respond more strongly prior to speech during motor preparation (as depicted in Reviewer 1-Question 4 responding to word-reading in a control task). While this new data suggests the involvement of dPreCG in auditory processing, we believe the magnitude of the response taken together with the response enhancement we report in the manuscript during speech (**Fig 4**) suggests that the increased activity in dPreCG during delayed feedback is due to auditory error processing rather than purely sensory.

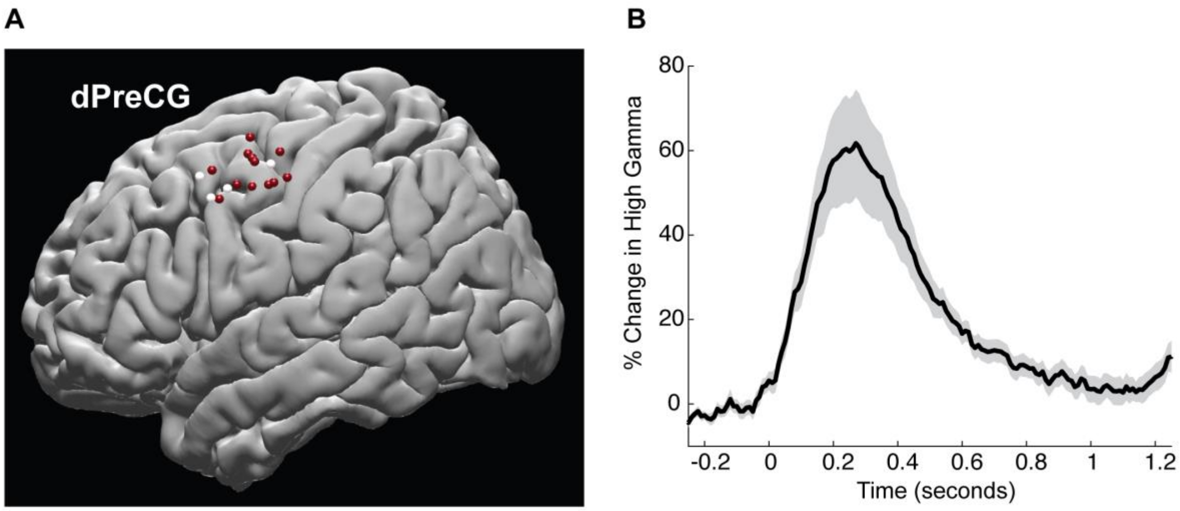

S2 Fig. Neural responses to a passive listening task in PreCG

2. The sensitivity to mismatch in Figure 3+4 is very interesting, but I have a few questions/clarifications and suggestions. This was calculated as a correlation coef, was this Person on Spearman? Spearman would be better as the feedback delay magnitude is not normally distributed. Perhaps also some sort of linear regression would also give a sense of the magnitude of the relationship (i.e. increasing delay x2 results in in % change in response). You state that the effect was larger in sentence than words, which appears to be the case in Fig 3G, but some stats would be useful, as would perhaps a histogram to get a sense of the distribution. You do this for the individual areas in Fig 4G, but an overall would be helpful. Also in 4G, you run the stats using unpaired t-tests, but since the same electrode is being compared in two conditions, shouldn't it be a paired t-test? (there are several other areas where an unpaired test is used, when a paired is likely more appropriate since the two samples are not independent).
The sensitivity to mismatch was calculated as the Spearman correlation between the neural activity and delay condition across trials. We thank the reviewer for pointing this out. We now indicated the type of correlation analysis in the manuscript.

As the reviewer suggested, we also performed a linear regression analysis by sorting the trials with respect to delay condition (no delay, 50, 100 and 200), calculating the mean high gamma activity for each trial and fitting a regression across trials for each electrode. As a measure of sensitivity, we show the slope values for each electrode for the word-reading and the sentence-reading tasks. The anatomical distribution of slope values showed a strong resemblance to the distribution of Spearman correlation values (**Fig 3E-F**). Moreover, sensitivity to DAF measured using slope values was significantly larger for sentence-reading task compared to word-reading task (Paired t-test: $t=2.3$, $p=0.02$). We now include these new results in the *Supporting Information* (**S4 Fig.**).

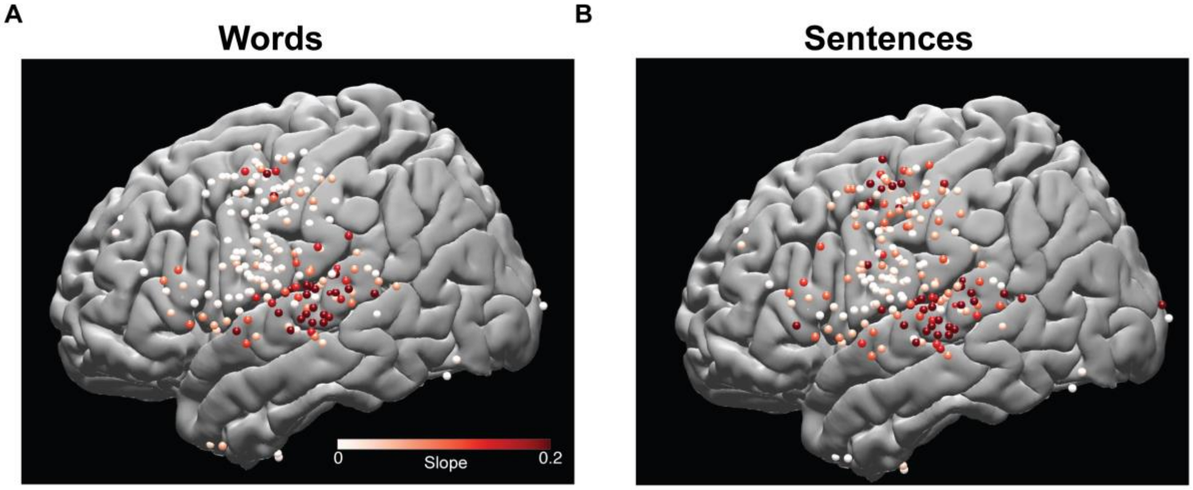

S4 Fig. Sensitivity to DAF calculated by linear regression

As per the reviewer's suggestion, we have now included a histogram (**Fig 3G**, also shown below) to demonstrate DAF sensitivity (measured as Spearman correlations) across all regions, and show that a higher DAF sensitivity in a larger number of electrodes was observed for the sentence-reading task compared to the word-reading task (paired t-test: t=11.15, p=8.3x10^{-24}).

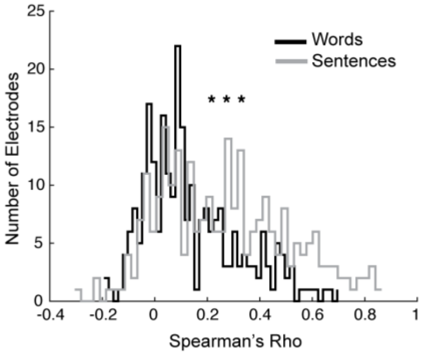

We also corrected the statistical analysis that was used to compare DAF sensitivity in individual regions by performing a paired t-test instead of the previous unpaired t-test (STG: t=6.4, p=1.4 x 10^{-7}; vPreCG: t=5.3, p=1 x 10^{-5}; dPreCG: t=8.3, p=2.7 x 10^{-9}; postCG: t=5, p=6.4 x 10^{-6}; SMG: t=2.3, p=0.03; IFG: t=4.5, p=4 x 10^{-4})

3. Fig 4H suffers from a large multiple comparisons problem. As I understand it, you did an ANOVA at each time point independently, and that is what is being shown. You should correct for the repeated calculations over time. My suggestions would be a FDR correction. Also, this shows the overall ANOVA output for all conditions, but it would be interesting to know how this changed for the different delays (i.e. the individual pairwise comparisons resulting from the ANOVA), to see if the onset time was dependent upon specific delays. There is a hint this might be the case in Fig 3C+D. The STG onset was 80ms on average, but was it different for a 50 vs 100 vs 200 delay?
We abandoned the permutation test for determining a significance threshold for the F-values. Instead, we now adjust the p-values using an FDR correction (q=0.05) and use a significance

threshold of p<0.001 to mark the time intervals when the neural response diverged significantly. In postCG, we now find a brief period between 110 and 440 ms at which neural responses diverge significantly, however this period does not reflect a neural response enhancement with increasing delays.

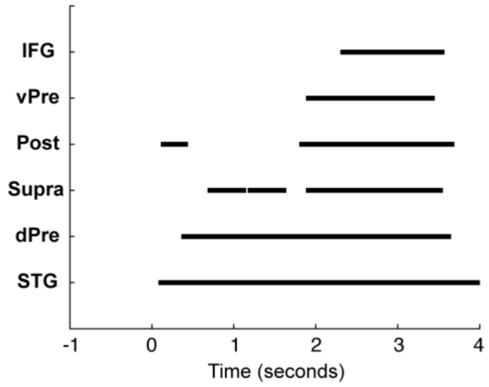

As requested by the reviewer, we then performed post hoc pairwise comparisons (FDR corrected at p = 0.05 and p<0.01) to test whether the divergence onset was different for different delays. As shown in the figure below, for STG, divergence onset occurred earliest for 0 vs. 100 at 240 ms after speech onset (indicated using red text). For 0 vs. 200, divergence occurred at 330 ms. And for 0 vs. 50, divergence occurred much later at 2.17 s. When we compared the 0 versus 200 ms delay conditions, the onset of divergence followed the same order as when we compared all four conditions: divergence onset occurred the earliest in the STG at 330 ms, then in dPreCG at 380 ms, in SMG at 660 ms, in postCG at 1.73 s, in vPreCG at 1.86 s and finally in IFG at 2.29 s. We now include these new results in the *Supporting Information* (**S5 Fig.**).

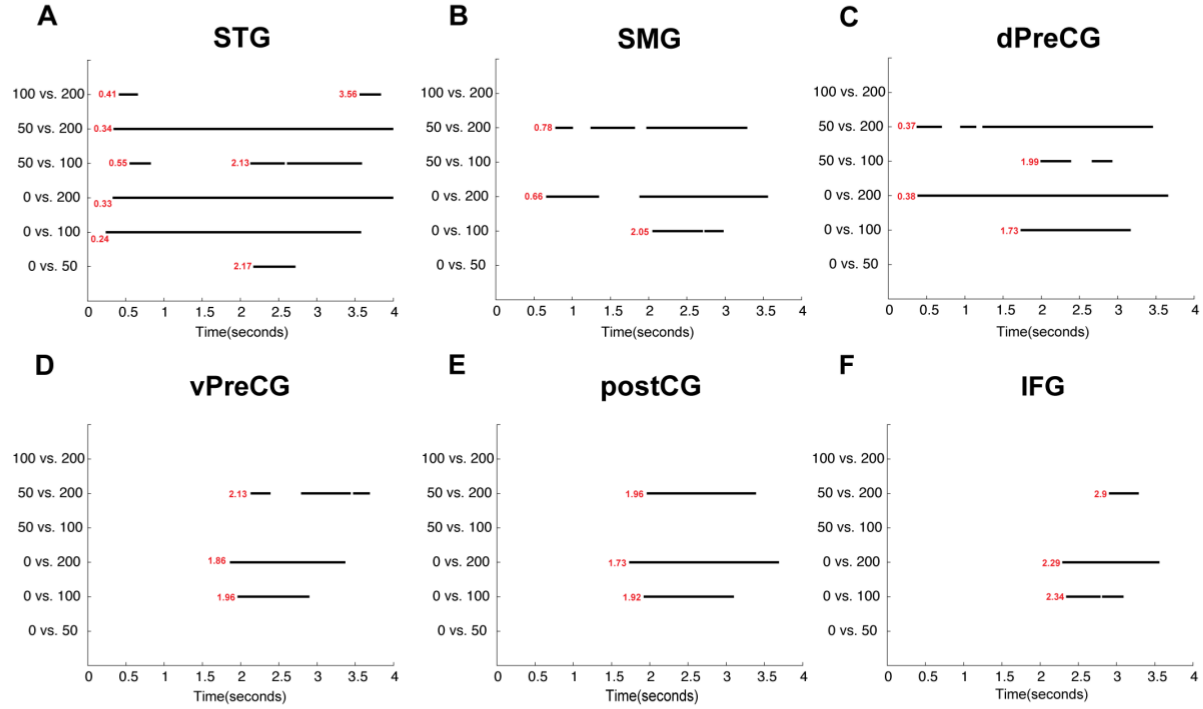

S5 Fig. Pairwise comparisons of neural responses to different delay conditions

4. After correcting for the speaking duration through DTW, was the response duration now longer during DAF?

We calculated neural response duration after DTW, by computing the time difference at full width quarter maximum of the response curve. We then compared the response duration for no delay and 200ms delay conditions using a paired t-test. Only for STG, the neural response duration was marginally larger for 200ms delay after DTW (STG: t=2.2, p=0.03). For the rest of the regions, there was no significant difference in neural response durations cofirming a successful alignment (vPreCG: t=1.3, p=0.2; dPreCG: t=1.7, p=0.1; postCG: t=1.2, p=0.25; SMG: t=1.6, p=0.14; IFG: t=0.3, p=0.8). These results are now added to the manuscript.

5. I am still a little uncertain that the dPreCG response is reflects feedback error as much as it does a motor signal. The lack of changes in words vs sentences would be more consistent with a motor hypothesis. The DTW might argue for error, but that primarily corrects for duration of response being dependent on duration of speech. It would be interesting to know how magnitude of response varied trial to trial with changes in speaking duration. Since you have the same sentences used under different delays, it should be possible to z-score the speaking durations relative to no-delay, and then see if there is any correlation of activity with this duration change (independent of delay magnitude). If these correlations were near zero, I would be more convinced that it's the feedback error and not a motor command signal.

We agree with the reviewer that it's difficult to dissociate the effect of feedback delay on the neural response from the effect of articulation duration. Articulation duration is longer for larger delays, and when articulation duration is longer, the neural response is longer. We tried correlating the increase in articulation duration (relative to no delay) with the neural response, however this analysis was very susceptible to the width of the time window across which we averaged the neural activity for each trial. To circumvent these issues, and provide a robust measure, we tried a different approach in which we either controlled for articulation duration or for the amount of feedback delay and then tested differences in neural responses.

To control for articulation duration, we identified all no delay and 200 ms delay condition trials of the same item that match in articulation duration (i.e. articulation duration difference is smaller than 10 ms). We compared the paired neural responses by performing a paired t-test at each time point. We corrected for multiple comparisons using FDR (q=0.05) and marked the time intervals that show a significant difference (p<0.01) for at least 200 consecutive milliseconds. As shown in **Fig 6A-F** (and copied below) we found that neural responses were enhanced in STG, dPreCG, SMG and IFG for DAF even when articulation durations were nearly identical, thus ruling out a purely motor articulatory account. Indeed, neural responses were not enhanced in vPreCG and PostCG where responses are presumably motor in nature and should not differ once controlled for articulation duration.

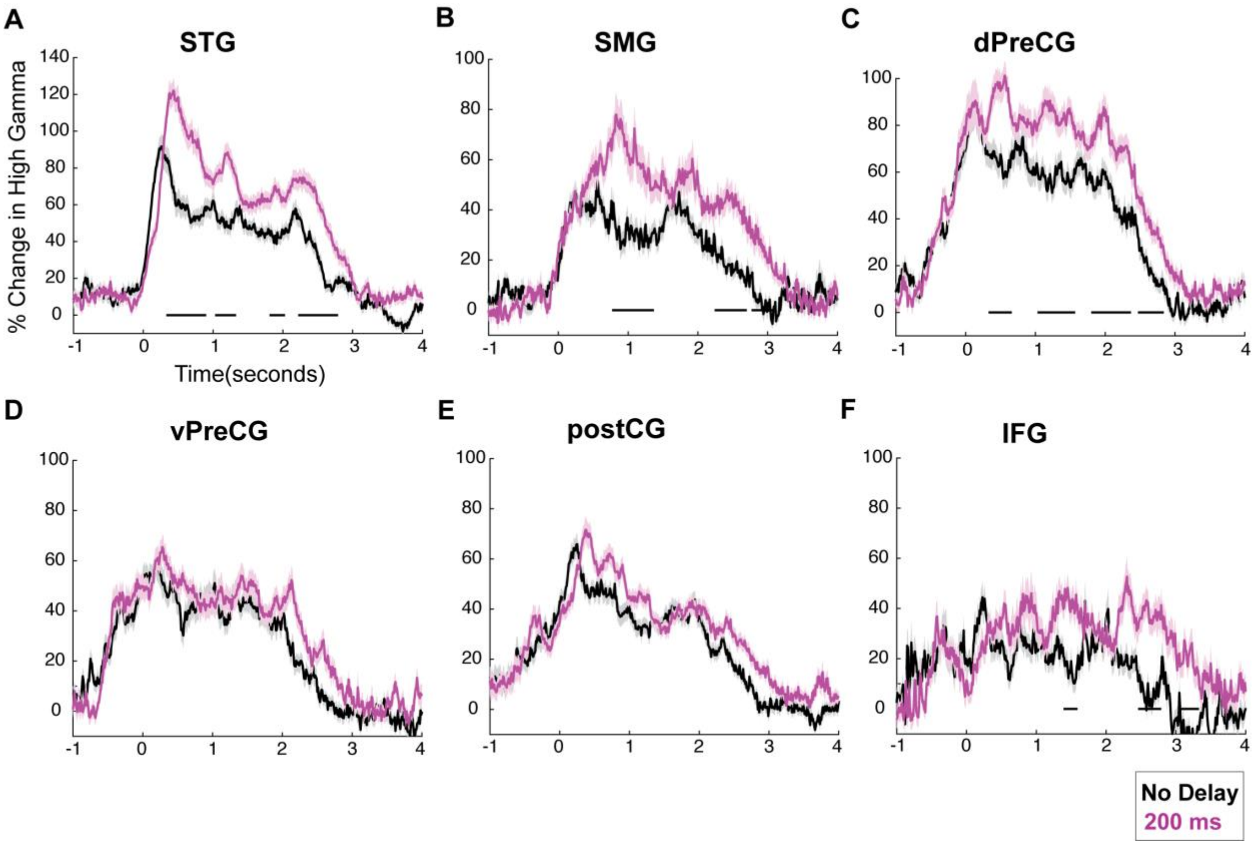

Figure 6. Neural responses for DAF sentence-reading task after controlling for articulation duration

To control for the amount of feedback delay, we split the 200 ms condition trials into 4 groups based on articulation duration: 0-25 percentile, 25-50 percentile, 50-75 percentile and 75-100 percentile. We compared the neural responses by performing a one-way ANOVA at each time point. We corrected for multiple comparisons using and FDR test (q=0.05) and marked the time intervals that show a significant difference (p<0.001) for at least 200 consecutive milliseconds. As shown in **Fig 7A-F** (and copied below), we did not find a significant neural response amplitude enhancement for longer articulation durations in any of the regions. This shows that while neural response amplitude is enhanced as a function of delay condition as shown in the manuscript, there is no such amplitude increase for longer articulations in the first several seconds during which all percentile conditions actually contain speech stimuli (e.g. the subject is still speaking). These complementary results eliminate the possibility that neural response enhancement is due to longer articulation duration and provide strong evidence that it is due to auditory error processing in these regions.

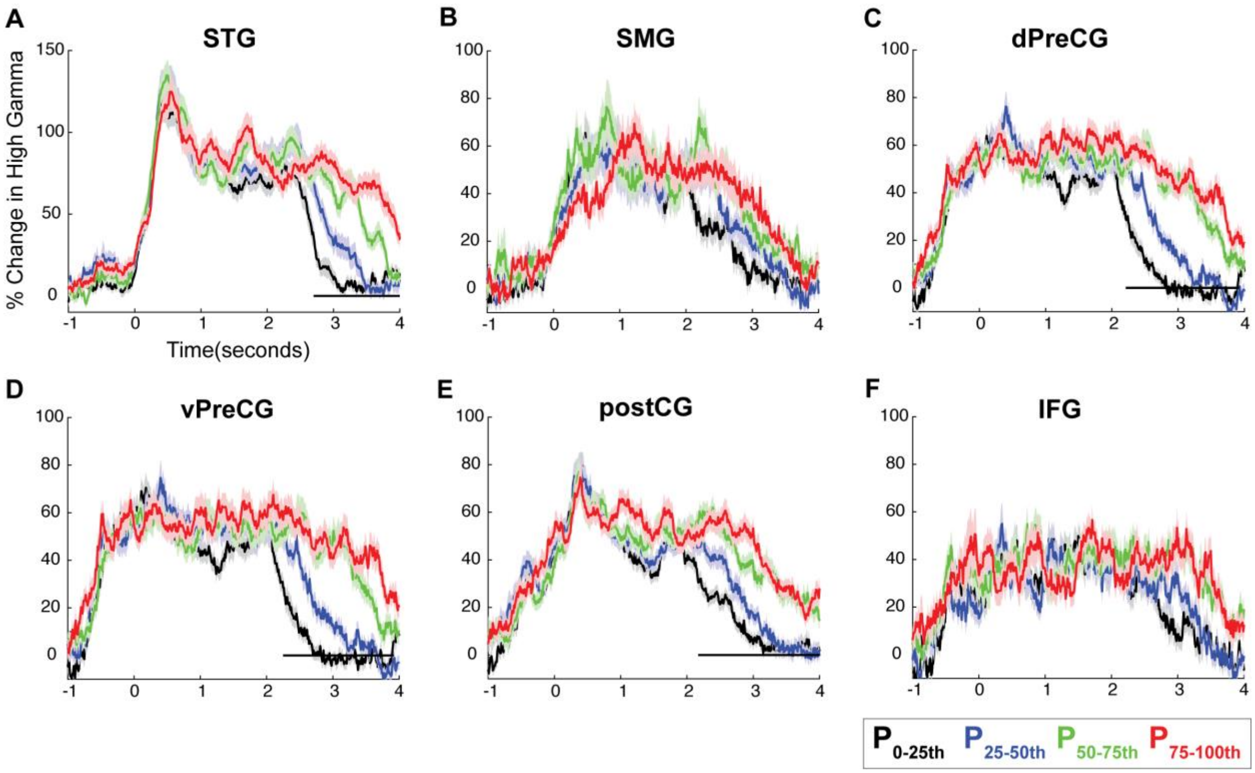

Figure 7. Neural responses for DAF sentence-reading task after controlling for delay condition

6. In the DTW analysis, it looks like a big delay was introduced. In Fig 5A, the uncorrected responses began right at 0, but the DTW for STG now begins at 1 second. Is this intentional or some sort of plotting error? Stats on the DTW were again done with an unpaired t-test rather than a pair (since the same site being compared in two different delays). Also for the DTW, was it done on the whole sentence, or separately on individual words and intervals within the sentence? Its not clear to me whether the overall change in sentence duration was due to changes in the words or the inter-word intervals. The DTW methods could use a bit more explanation in general.

We thank the reviewer for pointing this issue out, there was indeed a plotting error when showing the warped time values on the x-axes. We made a mistake when converting warping paths to time in seconds. We now corrected this error and updated the plots.

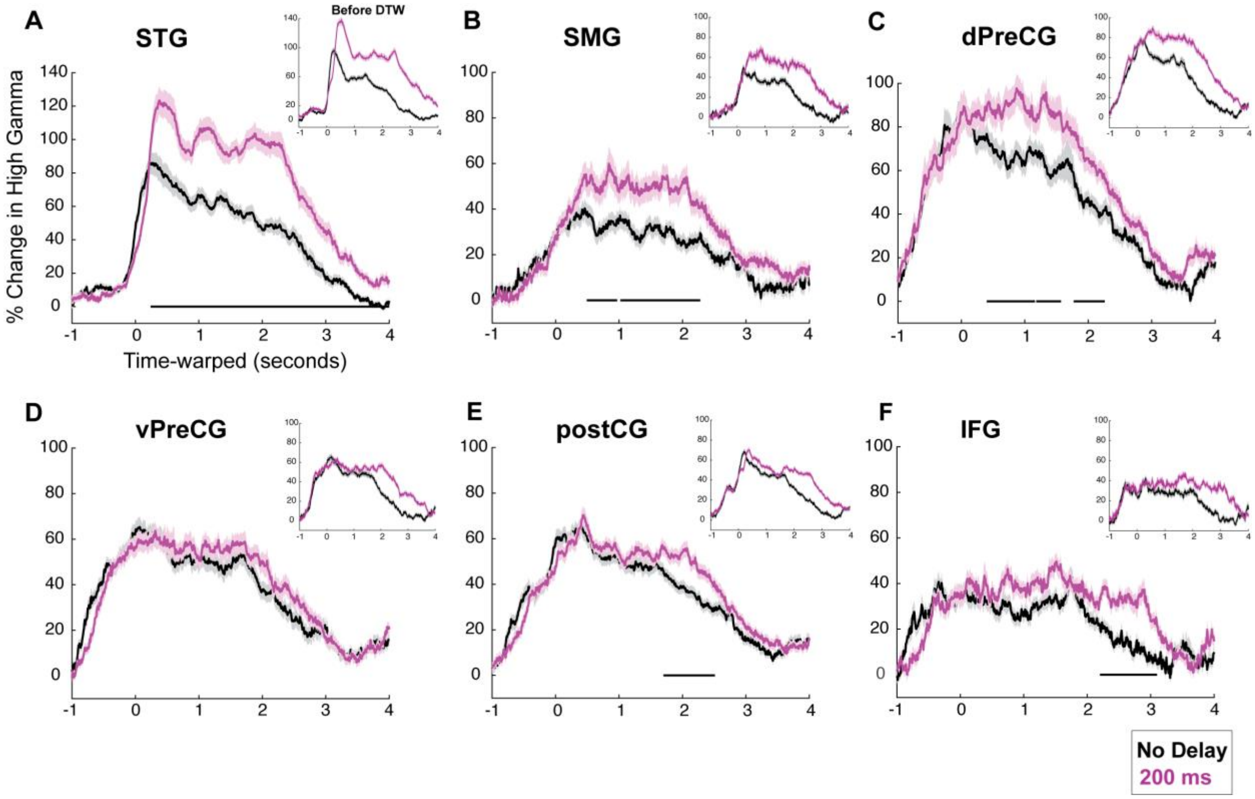

Figure 5. Time warped neural responses during sentence-reading with DAF

To compare the neural responses after DTW, we now performed a paired t-test at each time point, corrected for multiple comparisons using FDR (q=0.05) and marked the timepoints when the response to no delay and 200 ms delay conditions were significantly different (p<0.01) for at least 200 consecutive milliseconds.

DTW was performed on the whole sentences. We did an additional analysis to test whether the overall change in sentence duration was due to prolonging words or pausing longer between the words. We ran a one-way ANOVA using either the word duration or the pause durations as a factor and subjects were introduced as a random factor. We found that both word duration F=13.97, p=0.002) and pause duration (F=14.81, p=0.002) increased with DAF similarly. These results justified using the total articulation duration (duration of words + duration of pauses) as the behavioral measure.

To explain DTW analysis in more detail, we now write in the manuscript:
"In the sentence-reading task, 6 different sentences (e.g. Sentence #1: "The cereal was fortified with vitamins and nutrients") were presented. Dynamic time warping (DTW) analysis was performed separately for the 6 different sentence stimuli. First, the speech spectrogram was averaged across frequencies for each sentence stimuli. Then, the mean spectrograms were averaged across trials of the same sentence stimuli (e.g. trials in which Sentence #1 was presented with no delay.) Then, DTW is performed to compare the averaged spectrograms for no delay and 200ms delay conditions (e.g. Sentence #1 with no delay versus Sentence #1 with 200ms) and the resulting warping paths were applied to the neural response signal for each trial. Finally, the transformed neural responses were averaged across trials for each sentence stimuli.

This procedure was performed to compare two conditions that resulted in the largest neural response difference (no delay versus 200 ms delay)."

7. For the comparisons of behavior across delays, did the ANOVA account for repeated measures of individual subjects?
Previously, we did not account for repeated measures of individual subjects and we found that articulation duration significantly increases with delay only for sentence-reading task. To address the reviewer's suggestion, we performed a repeated measures ANOVA by introducing subjects as a factor. We now find that articulation duration increases with delay both for word-reading ($F=7.76$ $p=0.015$) and sentence-reading tasks ($F=20.54$ $p=0.0005$). We now include these results in the manuscript.

8. In the discussion, you may also want to comment on even longer delays, as the old 1950s data suggested a peaked behavioral effect of delay, increasing to 200ms, then decreasing again. This raises many interesting possibly hypotheses.
We now added the following paragraph in the discussion section:

"Our results showed that the maximal disruption of speech occurred at 200 ms feedback delay for both word-reading and sentence-reading tasks. Speech paradigms in previous DAF studies used various amounts of delays ranging from 25 to 800 milliseconds and consistently reported that the strongest disruption of speech occurred at 200 millisecond delay (Lee 1950, Black 1951, Fairbanks 1955, Stuart, Kalinowski et al. 2002, Yamamoto and Kawabata 2014). This time interval is thought to be critical for sensorimotor integration during speech production because it is of about the same order of average syllable duration. Given that the temporal distance between two consecutive stressed syllables is roughly 200 milliseconds, it has been suggested that delaying auditory feedback by this amount of time causes a rhythmical interference that results in the maximal disruption of speech fluency (Kaspar and Rübeling 2011)."

9. Few questions about the feedback hardware. How much was the feedback signal amplified relative to microphone signals? I am also confused on the paradigm. Was the delay done in blocks, with several reps (that was suggested in the methods, but unclear) before changing the delay?
To amplify the feedback signal relative to the microphone signal, we used the Psychtoolbox function PsychPortAudio ('Volume') by setting the audio output to 10, which yielded an actual 30% amplification.

Feedback delay was not introduced in blocks. We presented different amount of delays randomly. We now write in the manuscript: "Trials with different amount of feedback delays (18 to 60 repetitions for each delay) were presented randomly with at least a 1 second inter-trial-interval."

<u>Minor points:</u>

1. Abstract: 'is generated to correct the estimate and subsequent motor commands' just reads a little awkward to me.

We changed this sentence to: “When actual feedback differs from this internal estimate, an error signal is generated to correct the internal estimate and update necessary motor commands to produce intended speech.”

2. Results, first paragraph: 'voice onset was delayed' (page 5). It was feedback delayed not voice onset (which evokes VOT, something completely different).
We corrected this ambiguity and now we write: “Subjects (N=15) performed a word-reading task (single 3-syllable words) while the auditory feedback of their voice was delayed (no delay, 50, 100 and 200 ms) and played back to them through earphones in real time, a paradigm known as delayed auditory feedback (DAF).”

3. In the discussion "The exquisite resolution" seems over-stated (page 12). Clearly, ECoG has great temporal resolution (compared to say fMRI), crappy spatial resolution, unless your comparison is scalp EEG.
We know write: ECoG recordings provided us with the precise spatiotemporal evolution of feedback processing in these distinct regions.”

4. Methods, in the discussion of bandpass filtering not sure where the dash is supposed to be in '0.01682.67' (page 17)
We corrected this to 0.01-682.67 Hz.

Reviewer #3: This electrocorticography (ECoG) study investigates neural substrates underlying the auditory feedback control of speech by using a delayed auditory feedback (DAF) paradigm. Although their data are very interesting because they obtained data using the high resolution ECoG, there is one major problem. It is that the study did not control the duration confound.

They used 4 different amounts (no delay, 50, 100, 200ms) for DAF. The behavioral results indicated that articulation duration increased significantly with delay. The longer the delay time, the longer participants articulate and listen to their own voices. The neural response differences they found could be caused by different duration associated with different delay amounts. A recent DAF study using EEG recordings includes a listening condition (passive listening to a recording of one's own voice) [1]. In this way, authors can control neural response differences associated with different durations of auditory stimuli. If they can run additional experiments including the listening condition, their results would become more reliable. Moreover, in order to prove the involvement of the dPreCG in error signal production, they should include a simple (without DAF) articulation condition that articulate sounds that match with durations of speech produced under different DAF conditions.

1. Toyomura A., Miyashiro D., Kuriki S, Sowman P. F. Speech-Induced Suppression for Delayed Auditory Feedback in Adults Who Do and Do Not Stutter. Frontiers in Human Neuroscience. 2020; 150.

We agree with the reviewer that it’s critical to control articulation duration to reveal neural response differences that are caused mainly by auditory error processing. ECoG recordings provide strong signal to noise ratio, which allows us to measure a robust neural response at each

single trial. Leveraging this advantage of ECoG recordings, we identified no delay and 200 ms trials that match in articulation duration (articulation duration difference is smaller than 10 ms) and compared the neural responses for these trial pairs.

To compare the neural responses, we performed a paired t-test at each time point, corrected for multiple comparisons using and FDR test (q=0.05) and marked the time intervals that show a significant difference (p<0.01) for at least 200 consecutive milliseconds. As shown in **Fig 6A-F** (and copied below), we found that neural responses were enhanced in STG, dPreCG, SMG and IFG for DAF even when articulation durations were similar. Neural responses were not enhanced in vPreCG and PostCG. These new results eliminate the possibility that neural response enhancement is due to longer auditory stimulation caused by longer articulation duration. They provide further evidence that the neural response enhancement is due to auditory error processing in these regions.

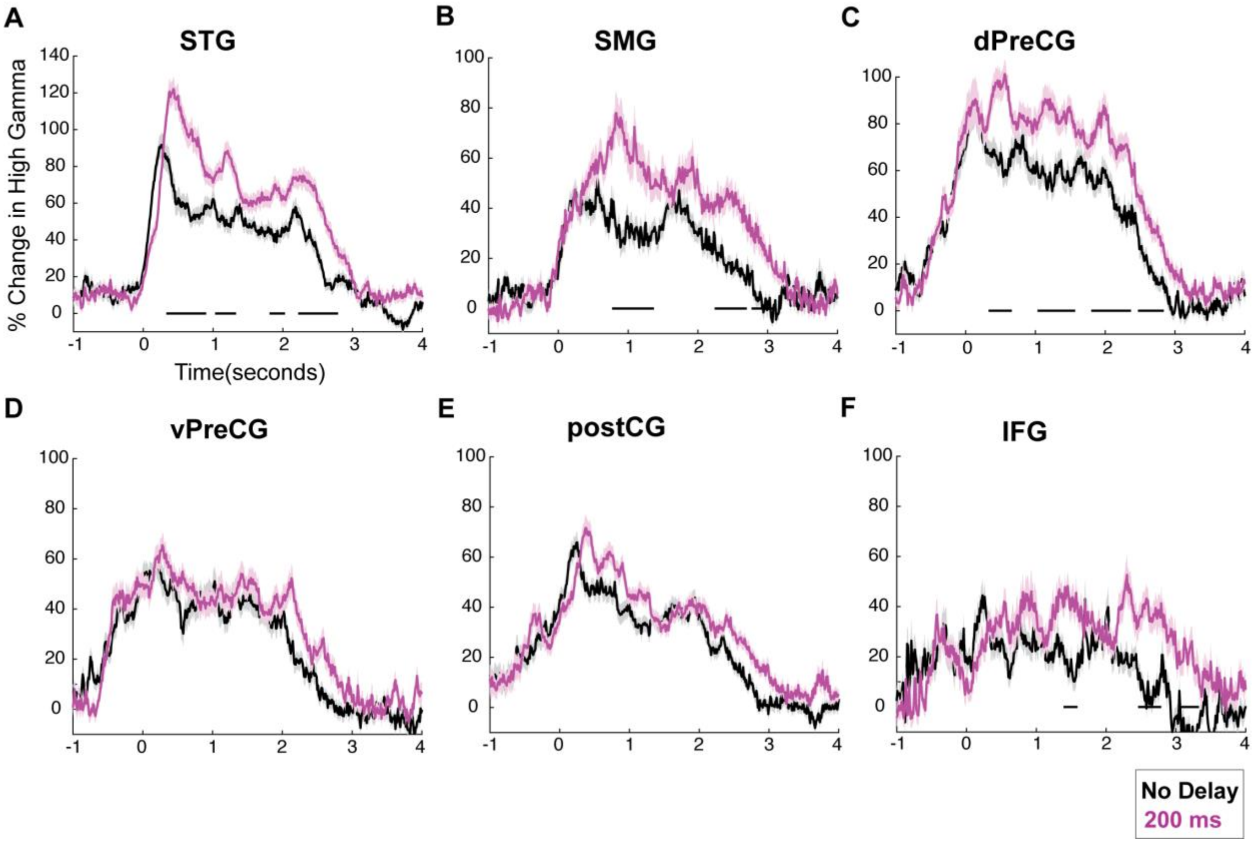

Figure 6. Neural responses for DAF sentence-reading task after controlling for articulation duration

References:

Chon, H., Kraft, S. J., Zhang, J., Loucks, T., & Ambrose, N. G. (2013). Individual variability in delayed auditory feedback effects on speech fluency and rate in normally fluent adults. *Journal of speech, language, and hearing research: JSLHR*, *56*(2), 489–504.

Flinker, A., Chang, E. F., Kirsch, H. E., Barbaro, N. M., Crone, N. E., & Knight, R. T. (2010). Single-trial speech suppression of auditory cortex activity in humans. *The Journal of neuroscience: the official journal of the Society for Neuroscience*, *30*(49), 16643–16650.
