# Supplementary material for: A cortical network processes auditory error signals during human speech production to maintain fluency
Source: PLoS Biol. 2022 Feb 3;20(2):e3001493. doi: 10.1371/journal.pbio.3001493 (PMC8812883; doi:10.1371/journal.pbio.3001493)
Supplement: Attachment — Submitted filename: Response to Reviewers_Oct21.docx [file pbio.3001493.s020.docx]

We greatly appreciate the positive comments and feedback of the editor and the reviewers. To address the remaining concerns, we now show additional analysis in our response below. We also added new figure panels to the revised manuscript (Fig 3C-E) and made changes in the discussion. Below, we provide a point by point response to reviewers:

Reviewer remarks:

Reviewer #1, Edward Chang: This manuscript has been significantly improved since the first submission. The responses from the authors are very helpful and have addressed most of my concerns. The newly added Figures. 6 & 7 provided important new evidence to support the sensitivity on auditory error in the dorsal precentral gyrus. The additional analysis included in the supporting information is also very helpful for me to understand the experimental details and controls. I appreciate the efforts the authors made to improve this work. I think the results presented in the current version are convincing and the work adds important information to the research of feedback processing in speech production. I have some remaining questions, but I believe they are all addressable.

(1) Fig. 3, is it possible to provide an example of a single electrode's activity, similar to Fig. 1D, since this is the first time to show the results for sentence reading? This also helps to illustrate the modulation of delay on the single electrode level, e.g. whether each delay change caused a similar amount of change in activity; how the activity evolves over time, etc.

We added a new panel to Figure 3 demonstrating neural responses for sentence-reading in single electrodes (same electrodes as in Figure 1C-E).

(2) Page 14, last sentence: the statement regarding "articulating longer and more complex speech stimuli" is not as grounded as it sounds (similar statements appear in multiple places). While I understand the results from sentence reading and its contrast to word reading, it is unclear whether it is the length or the complexity that drives the modulation in dPreCG. One possible control is to ask the subject to repeat the same word (or syllables) multiple times so that it matches the length of a sentence, or to use words with complex structure while keeping the number of syllables the same. Ideally one could test these in experiments, but I understand the difficulty with intracranial recordings. So if these data are not available, some discussions about the limitations need to be added.

Now we write in the discussion: “While our results ruled out the possibility that response enhancement in dPreCG during sentence-reading is due to longer articulation duration, we did not have the necessary control tasks to test whether stimulus length or complexity drives this modulation. However, dPreCG is well known to be involved in movement planning and execution [46, 47]. Previous studies reported increased activation in this region when subjects produced complex syllable sequences, suggesting a role in the planning and production of long speech utterances with appropriate syllable timing [48]. Considering that dPreCG activation occurred only when subjects produced sentences with DAF, we predict that it plays a critical role in maintaining prosody and speech fluency during the articulation of long utterances.”

(3) Fig. 6 & 7, while these are important new results, it is hard to know how much variation there is across individual electrodes. Is it possible to show a scatter plot (or other reasonable formats) to summarize the comparison for all the electrodes? Presumably, a window with the largest effect can be selected to quantify the difference across conditions. One potential concern for Fig. 7 is that the activity of some electrodes is positively correlated with articulation duration, and the activity of other electrodes is negatively correlated. When averaged together, it appears that there is no modulation.

To show the variation across electrodes in Figure 6, we averaged the high gamma response in each electrode between 0 to 2 seconds and made a scatter plot showing averaged responses for ‘No delay’ versus ‘200 ms delay’ conditions. The figure below shows the scatter plots for different regions where each circle indicates an electrode. A majority of the electrodes in STG, SMG and dPreCG showed larger neural responses for 200 ms delay. We compared the neural responses for ‘No delay’ versus ‘200 ms delay’ conditions across electrodes and only STG, SMG and dPreCG showed significantly larger neural responses (paired t-test; STG: t=5.6, p=2x10^-6^, SMG: t=2.2, p=0.04, dPreCG: t=2.43, p=0.02, vPreCG: t=0.39, p=0.7, postCG: t=0.86, p=0.4, IFG: t=2.03, p=0.06).

To address the reviewer’s concern about Figure 7, we show the neural responses for different articulation durations in every single dPreCG electrode in the below figure. Neural response amplitudes are similar for different articulation durations in all the electrodes.

(4) In the abstract, "response enhancement in dPreCG occurred only when subjects profoundly slowed down their speech" seems misleading. This implies that dPreCG activity is modulated by articulation (speed/duration), which is the opposite of what the main conclusion is. In fact, the authors have shown that given the same delay, articulation duration does not change the dPreCG activity level. Please consider changing the expression.
We now write in the abstract: “Critically, response enhancement in dPreCG occurred only during articulation of long utterances, which causes a continuous mismatch between produced speech and reafferent feedback.”

Reviewer #2: I think overall, the authors have done a good job addressing most of my comments.
One of my concerns still remains about auditory controls.  While the authors did show some speech playback data as a supplement for their revised manuscript, there are still some problems since it was 1) not the subjects own voice, and 2) did not test the effects/distortions from feedback delays.  I think its probably fine, but may need some more caveats in the discussion.

We did not have an auditory control task to play subjects’ own distorted voice back to them, however a previous EEG study by Toyomura et al. performed this exact experiment. They analyzed auditory evoked potentials (from three midline electrodes Fz, Cz and Pz) while the subjects listened to their own recorded voice, which included both the directly vocalized sound and the delayed sound. They found that the N1 amplitude of the auditory evoked potentials decreased as the delay increased. This is because for short delays, the directly vocalized sound and the delayed sound overlapped and produced a larger sound density, which in turn caused a larger auditory response. On the other hand, for the speaking condition, they found that the N1 amplitude increased as the delay increased reflecting an auditory error signal, which is in line with our findings. Using ECoG, we were able localize the exact network components that produce these auditory error signals, which surprisingly involved dPreCG.

- Toyomura, A., Miyashiro, D., Kuriki, S., & Sowman, P. F. (2020). Speech-Induced Suppression for Delayed Auditory Feedback in Adults Who Do and Do Not Stutter. *Frontiers in human neuroscience*, *14*, 150. https://doi.org/10.3389/fnhum.2020.00150

We now write in the discussion by citing Toyomura et al.: “A possible control, which we were unable to complete in this study, would be to replay a recording of the subjects’ own delayed voice back to them [36] to completely eliminate the possibility of a purely sensory explanation for the observed response enhancement in dPreCG. But it is note-worthy that in addition to responding to auditory speech stimuli, dPreCG electrodes also respond strongly prior to speech during motor preparation. This pre-articulatory activity taken together with the increased activity during delayed feedback suggests auditory error processing rather than a purely sensory explanation. We predict that dPreCG may be the hub for generating the internal estimate of the speech output by processing both the efference copy and auditory error signals during speech production.”

Reviewer #3: Thank you for revising your paper to respond to my comments. I think this paper is ready to be published in Plos Biology.
